# A comprehensive review direct methods to overcome the limitations of gas injection during the EOR process

**DOI:** 10.1038/s41598-024-58217-1

**Published:** 2024-03-29

**Authors:** Masoud Shafiei, Yousef Kazemzadeh, Mehdi Escrochi, Farid B. Cortés, Camilo A. Franco, Masoud Riazi

**Affiliations:** 1 IOR/EOR Research Institute, Enhanced Oil Recovery (EOR) Research Centre, Shiraz University, Shiraz, Iran; 2 Department of Petroleum Engineering, Faculty of Petroleum, Gas, and Petrochemical Engineering, Persian Gulf University, Bushehr, Iran; 3 Grupo de Investigación en Fenómenos de Superficie, Departamento de Procesos y Energía, Facultad de Minas, Universidad Nacional de Colombia, Sede Medellín, 050034 Medellín, Colombia; 4 School of Mining and Geosciences, Nazarbayev University, Kabanbay Batyr 53, 010000 Astana, Kazakhstan

**Keywords:** Enhance oil recovery, Formation damage, Mobility ratio, Asphaltene deposition, Gas injection, Gas-phase modification, Engineering, Chemical engineering

## Abstract

Among the Enhanced Oil Recovery (EOR) methods, gas-based EOR methods are very popular all over the world. The gas injection has a high ability to increase microscopic sweep efficiency and can increase production efficiency well. However, it should be noted that in addition to all the advantages of these methods, they have disadvantages such as damage due to asphaltene deposition, unfavorable mobility ratio, and reduced efficiency of macroscopic displacement. In this paper, the gas injection process and its challenges were investigated. Then the overcoming methods of these challenges were investigated. To inhibit asphaltene deposition during gas injection, the use of nanoparticles was proposed, which were examined in two categories: liquid-soluble and gas-soluble, and the limitations of each were examined. Various methods were used to overcome the problem of unfavorable mobility ratio and their advantages and disadvantages were discussed. Gas-phase modification has the potential to reduce the challenges and limitations of direct gas injection and significantly increase recovery efficiency. In the first part, the introduction of gas injection and the enhanced oil recovery mechanisms during gas injection were mentioned. In the next part, the challenges of gas injection, which included unfavorable mobility ratio and asphaltene deposition, were investigated. In the third step, gas-phase mobility control methods investigate, emphasizing thickeners, thickening mechanisms, and field applications of mobility control methods. In the last part, to investigate the effect of nanoparticles on asphaltene deposition and reducing the minimum miscible pressure in two main subsets: 1- use of nanoparticles indirectly to prevent asphaltene deposition and reduce surface tension and 2- use of nanoparticles as a direct asphaltene inhibitor and Reduce MMP of the gas phase in crude oil was investigated.

## Introduction

**Figure 1:**
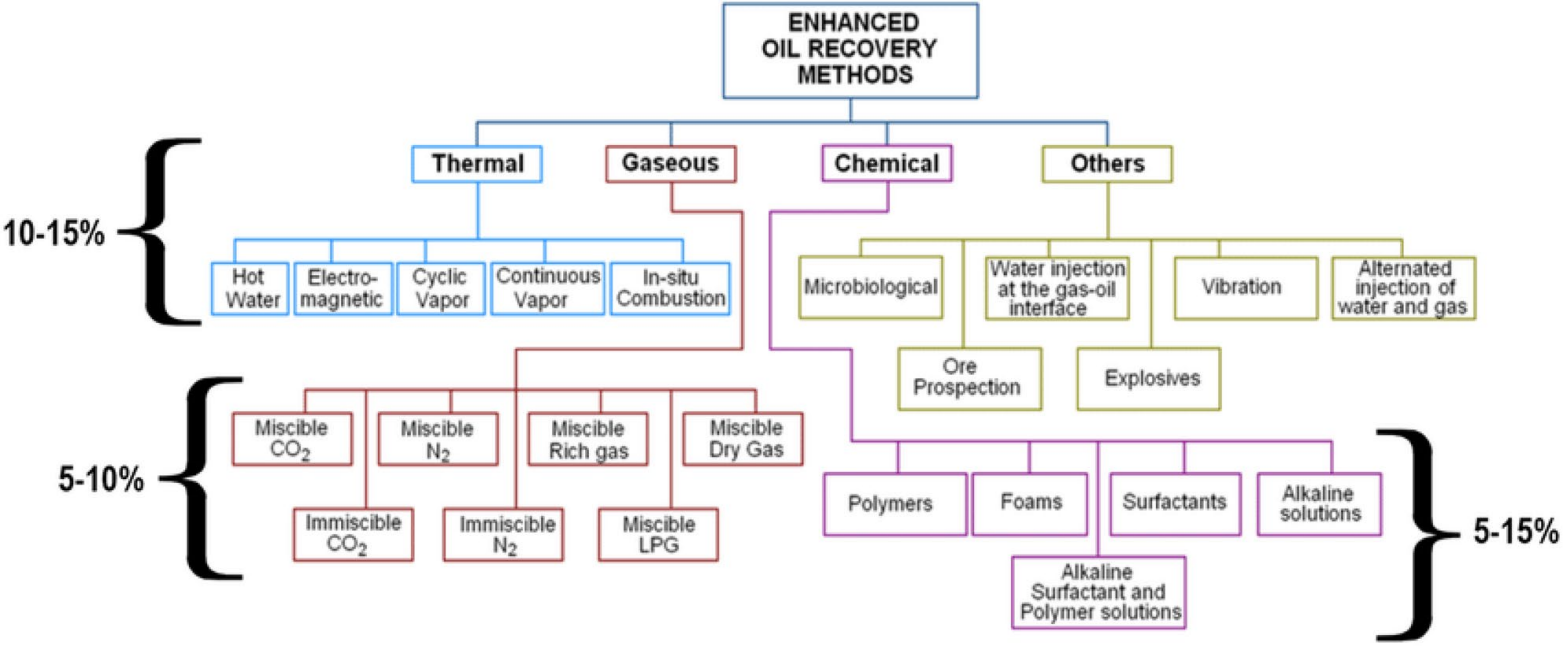
Summary of EOR methods^12^.

Due to the growing world population, and increasing incomes of developing countries, the need for energy, especially hydrocarbon resources (oil and gas) is strongly felt^1–3^. According to studies, after secondary recovery, an average of two-thirds of the oil remains in place in the reservoir^4–7^. As a result, due to limited hydrocarbon resources and high costs of exploration and development of new fields, this amount of residual oil should be produced as much as possible by Enhanced Oil Recovery (EOR) processes^8–10^. EOR methods are very wide and varied but are generally divided into five categories: water-based, gas-based, thermal, microbial, and other EOR methods (Fig. 1)^11,12^.

**Figure 2:**
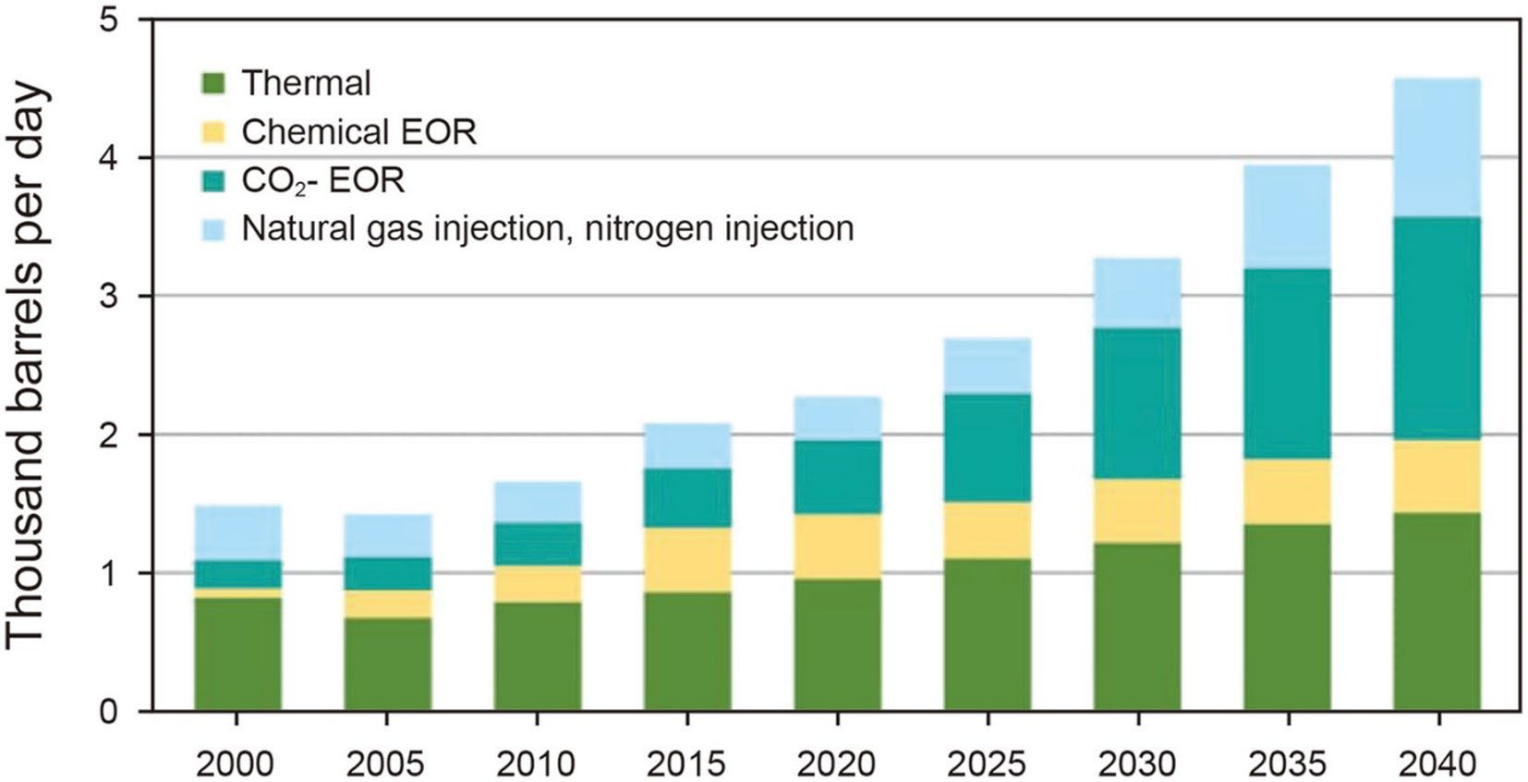
The efficiency of EOR projects and their prospects by year^13^.

The efficiency of EOR projects (from the point of view of oil production) has been carried out and their prospects with these methods are shown in Fig. 2.

As shown in the Fig. 2, gas injection is one of the most popular EOR methods and has the largest share in recent years. Among the gases, carbon dioxide gas has received a lot of attention because it is a greenhouse gas, and its injection into the reservoir, in addition to increasing the oil production, is an environmentally friendly work. In addition, carbon dioxide gas is cost-effective and has a lower MMP than other gases. Gas injection into the reservoir can increase oil production by reducing surface tension, and oil swelling, and increasing or maintaining reservoir pressure (reservoir energy)^14–16^. At present, gas injection, especially carbon dioxide, is the most popular method for EOR from sandstone and carbonate reservoirs (with low to medium molecular weight oils)^17–19^. Gas injection, as mentioned above, is done in the oil field for two reasons: 1-Maintaining the reservoir pressure 2-enhancing oil recovery by reduction of viscosity and oil swelling. Gas injection (rich and lean gases, flue gas, nitrogen, and carbon dioxide) can increase recovery by 10% or even more^11,20–24^. The greatest increase in recovery occurs when the gas is injected in miscible conditions. The two-phase miscibility process (gas and oil) occurs due to multiple contacts between the two phases. To achieve this, the surface tension must be close to zero. By creating these conditions, the system will no longer be two-phase and we will have only one phase, and thus the recovery efficiency will reach its maximum^24^.

In general, gas injection mechanisms are divided into three categories: miscible injection, near miscible injection and immiscible injection. Immiscible flooding is considered when dealing with heavy oil reservoirs because in such reservoirs, due to the high viscosity of the oil, its movement in the pores of the rock becomes extremely difficult, and the remaining oil increases. As a result, by immiscible injection and by two effective mechanisms: (1) oil inflation and (2) reduction of oil viscosity, the sweep efficiency, and residual oil can be increased and decreased, respectively. However, it should be noted that the best reservoirs for this type of injection are heavy oil tanks with a maximum of 30 API and low pressure^25^. Among the three mentioned modes for gas injection, the most common is the miscible injection mechanism. In this case, the gas dissolves when it comes in contact with the fluid in the reservoir, affecting the oil–gas equilibrium. Changing the equilibrium between the phases causes a change in the behavior of the fluid and can be effective in three ways:

1. Evaporating mechanism means the penetration of light oil components in gas
2. Condensing mechanism means the penetration of heavy components of gas in oil
3. The combination of two mechanisms in which heavy components of gas are dissolved in oil and light components of the oil are dissolved in gas.

One of the parameters that strongly affects the miscible injection process is the minimum miscible pressure or MMP. The minimum miscible pressure is the minimum pressure at which the injected fluid is dissolved in the reservoir fluid. The minimum miscible pressure is a function of various parameters such as reservoir pressure, reservoir temperature, injection fluid composition, and reservoir fluid composition^26^.

The miscible injection process between gas and crude oil is done in two ways: (1) miscibility occurs in the first contact, (2) miscibility occurs at multiple contacts. The miscibility process in the first contact occurs when two phases are dissolved in all ratios to each other^27^. The multiple-contact miscibility process is divided into two mechanisms, which are: evaporating and condensing mechanisms. In the evaporating process, light or medium components of oil enter the gas phase and enrich it, and then during the evaporation process, the enriched gas dissolves in the oil, and a miscible zone is created between the two phases. The miscibility process improves with increasing pressure in both phases. However, this phenomenon in the gas phase has a greater impact on the dissolution process, which is due to the high compressibility of the gas phase^28,29^. As the pressure increases, the molecules become closer to each other, and two-phase interactions will increase. These interactions will improve the two-phase dissolution process. In addition to all these advantages, gas-based methods have enormous limitations and challenges that will be fully addressed in the next sections^30,31^.

The purpose of this article is to review the gas injection process, its challenges, and the solutions that have been proposed for these challenges. In the first part, an introduction to the gas injection process was provided. In the second part, the related challenges were examined. In the third section, gas thickeners were examined. In the fourth section, the effects of nanoparticles on asphaltene deposition and minimum miscible pressure were discussed. The use of nanoparticles in upstream oil and gas industries is divided into five general categories. (1) Using nanoparticles to improve the drilling fluid. For this purpose, nanoparticles can improve the rheological properties of the drilling fluid and in addition, they can perform better in creating a filter cake and reduce the penetration of the drilling fluid into the formation^32^. (2) Another application of nanoparticles is improving thermal conductivity in drilling fluid and controlling filtration. Filtration control prevents excessive penetration of drilling fluid into the formation, and improving thermal conductivity can prevent the increasing temperature of the drill bit^33^. (3) The third application of nanoparticles in the oil and gas industry is their use as insulation of fluids at high temperatures and conformance control^34–36^. (4) Nanoparticles are also used in cementing systems in oil and gas industries, which include self-healing, and nanosensors to improve well integrity^37,38^. (5) In addition to the mentioned applications, the nanoparticles application has been studied a lot due to their unique properties in increasing oil recovery or tracing properties. and finally, in the fifth section, a summary of these contents was presented.

## Challenges of gas injection

### Rock-fluid or fluid–fluid incompatibility

Fluid–fluid compatibility as well as rock-fluid compatibility is one of the important parameters that has always been considered by researchers and oil companies in designing the gas injection process. One of the important damages that occur during gas injection in the reservoir is asphaltene deposition due to a change in fluid composition. Asphaltene deposition in the reservoir, in addition to reducing permeability and porosity, can affect the amount of oil recovery by changing the wettability of the reservoir rock to oil-wet and reducing the efficiency of the EOR process^39–44^. Many researchers have studied this issue both on the laboratory and field scale. They showed that injecting gas into the reservoir as the EOR method could change the composition of the oil to cause asphaltene deposition and damage the formation, which ultimately reduces the production and permeability of the system^45–47^. Asphaltene precipitation and deposition can have many destructive effects on reservoir parameters such as permeability reduction by pore plugging, and wettability alteration to oil wet. In addition, asphaltene deposition reduces the viscosity of the oil due to the loss of asphaltene and can also cause to damage the equipment by deposition during the operation system, pipelines, separators, etc. And affect the oil production and raise concerns about safety, staff health, and the environment^48,49^.

In 2006, Moghadasi et al. investigated the process of formation damage during gas injection at the Kupal oil field in southwestern Iran (Saturates = 26.88% (w/w), Asphaltene = 0.66% (w/w), Resin = 4.97% (w/w), Aromatic = 67.99% (w/w)). They used the PVT test to start their studies to evaluate the amount of asphaltene deposition, and after performing this static test, they flooded the core with carbon dioxide. They showed that by injecting gas into the core, asphaltene deposition could reduce the permeability from 45 to 90% and practically cause the EOR process to fail^42^. Pak et al., at 2011 examined the oil production process under three different scenarios (GOR = 240.53 SCF/STB, reservoir oil Mw = 195% mol). Production processes included natural depletion, recycled gas injection, and carbon dioxide injection. Production scenarios were performed on a sandstone core, and asphaltene deposition was measured by measuring the asphaltene content at the inlet and outlet. The results showed that the lowest asphaltene deposition occurred due to natural depletion, but the highest asphaltene deposition was observed (indirectly) in the injection of recycled gas^50^. In 2018, Shen and Sheng studied the process of asphaltene deposition and reduction of permeability during the Huff and Puff injection of carbon dioxide (Density = 0.794 g/cm^3^ API Gravity = 46.7° API Viscosity = 3.66 cP at 69 °F). The results showed that the gas injection in the first period (first Huff) had the highest asphaltene deposition compared to other cases, and the most important stage of gas injection is in terms of asphaltene deposition. Finally, they stated that the highest amount of deposition occurs in the core plug^51^. Wang et al., investigated the process of asphaltene deposition during carbon dioxide injection (Saturates = 29.16% (w/w), Aromatics = 34.06% (w/w) ,Resins = 29.08% (w/w), Asphaltenes = 7.70% (w/w)). In this study, they injected miscible carbon dioxide into sandstone cores and measured the amount of asphaltene produced at different times. The results of Wang et al.’s study showed that the amount of asphaltene deposition decreases after the gas breakthrough, in other words, the asphaltene deposition is higher before the breakthrough^52^. Soroush et al., by examining the amount of asphaltene in the produced oil, investigated the process of asphaltene deposition during gas injection in a miscible and immiscible condition (Density of crude oil = 911.24 kg/m^3^, Viscosity = 370 cp, asphaltene content = 16.1% (w/w) at 22 °C). They showed that the amount of asphaltene deposition is lower at pressures lower than the miscible pressure and the amount of asphaltene deposition will increase with increasing gas injection pressure^53^. Zanganeh et al., conducted a comparative study on the amount of asphaltene deposition under the injection processes of nitrogen, carbon dioxide, and hydrocarbon gas (synthetic oil composition: Toluene = 75% (w/w), n-C7 = 23% (w/w), Aspahltene = 2% (w/w)). They found that carbon dioxide and methane increased asphaltene deposition while nitrogen (as an inert gas) did not significantly increase asphaltene deposition. In addition, it was observed that the amount of asphaltene deposition in contact with carbon dioxide is higher than methane. All tests were performed at a temperature of 90 °C and 15% mol of each gas at different pressures, which increased with the increasing pressure of asphaltene deposition^54^. In 2019, Qian et al., studied the deposition of asphaltene during carbon dioxide injection by the NMR method in three conditions: immiscible, near-miscible, and miscible (Saturates = 66.63% (w/w), Aromatics = 27.54% (w/w), Resins = 4.89% (w/w), Asphaltenes = 0.94% (w/w)). They observed that with increasing injection pressure, more light components were vaporized by carbon dioxide, and heavy oil components remained in the core sample and concluded that asphaltene deposition would increase with increasing injection pressure. They also stated that due to sufficient interaction between the two phases in larger pores (10–1000 ms) asphaltene deposition in these areas will be more than in micropores. They noted that asphaltene deposition, in addition to closing pores, also alters wettability. Simulation studies also show an increase in the amount of asphaltene deposition during the injection of various gases^55^. In 2019, Dashti et al., conducted a comparative study on the injection of different gases and the effect of gases on asphaltene deposition (Saturates = 38.74% (w/w), Aromatics = 50.59% (w/w), Resins = 6.17% (w/w), Asphaltenes = 4.25% (w/w)). In this study, carbon dioxide, nitrogen, and methane were used and at different pressures, the behavior of the system was examined by a microscope. They found that most of the asphaltene deposition occurred in the case of carbon dioxide injection, which was 1.2 times more than methane at 100 bar, and nitrogen did not significantly alter the asphaltene deposition. They also showed that asphaltene deposition increased with increasing pressure^56^. Cho et al. in 2021 used the simulation of injection of carbon dioxide alternating LPG with asphaltene deposition to investigate the efficiency of the EOR process (saturation pressure of oil = 20.339 kPa, Mw = 17.1 g/mol, API gravity = 19° API). They stated that simultaneous injection of LPG along with the injection of water alternating and carbon dioxide due to reduced surface tension, injection efficiency increases. But in addition to these positive effects, due to the change in the composition of the oil during LPG injection, the amount of asphaltene deposition increases, and the formation damage becomes more severe. A summary of case studies is provided in the Table 1^57^.

**Table 1:** Asphaltene deposition in oil reservoirs during gas injection.

Author	Type of gas	Fluid properties	Location	Results	References
Leontaritis et al.	CO2	Not mentioned	Little Creek, Mississippi	Asphaltene deposition during gas injection in the tubing	^58^
Leontaritis et al.	CO2	Bubble point pressure = 1250 psig. GOR = 850 Scf/bbl API Gravity = 28° API	southeastern Saskatchewan	Injection of carbon dioxide causes the deposition of asphaltene	^59^
Novosad and Costain	CO2	Asphaltenes = 5.5% (w/w), Wax = 10.45% (w/w) Others = 84.1 5% (w/w)	Ventura, California	The gas injection has caused the deposition of asphaltene	^60^
Negahban et al.	CO2 and hydrocarbon gas	API gravity = 39 ˚AP60I, GOR = 551 scf/bbl Asphaltenes = 0.94% (w/w) Saturates = 54.2% (w/w) Resins = 4.6% (w/w) Aromatics = 40.3% (w/w)	Abu Dhabi	Carbon dioxide injection did not affect asphaltene deposition but hydrocarbon injection increases asphaltene deposition	^61^
Gonzalez et al.	CO2, N2, CH4	Asphaltenes = 15.5% (w/w) Aromatics = 35.9% (w/w) Resins = 9.0% (w/w) Saturates = 39.2% (w/w)	Gulf of Mexico	The results showed that nitrogen, methane, and carbon dioxide had the greatest effect on asphaltene deposition, respectively	^62^
Rong-Tao et al.	CO2-WAG	Asphaltenes = 1.32% (w/w) Aromatics = 13.52% (w/w) Resins = 8.59% (w/w) Saturates = 63.78% (w/w)	Changqing	The results of this study showed that asphaltene deposition increases with an increased injection rate of water alternating gas	^63^

### Unfavorable mobility ratio

Another limitation of gas injection is the unfavorable mobility ratio. The mobility ratio during gas injection is defined as follows:

1 $$ mobility \;ratio = \frac{mobility\; of\; injected\; fluid}{{mobility\; of\; reservoir\; fluid }} $$

The mobility of each phase is equal to the effective permeability divided by the viscosity of that phase. The mobility ratio for the gas injection process to oil reservoirs during the EOR process is always unfavorable due to the very low viscosity of the gas phase. This phenomenon causes low efficiency of the gas-based EOR process, therefore, this parameter must be controlled to achieve the appropriate efficiency. The main reason for the unfavorable efficiency of the gas-based EOR process is the phenomenon of viscous fingering, which also cause an early gas breakthrough^64^. There are several ways to overcome this problem, which will be fully explored in the following sections, but in summary, so far, foam injection methods and water alternating gas injection have been most used in field processes and have been able to control the mobility ratio. However, there are methods such as polymer injection, surfactants, and thickeners that can increase the gas phase viscosity but have not yet been used in the field^65–72^.

## Gas mobility ratio control

There are various methods to enhance oil recovery from oil reservoirs, such as water-based methods (injection of nanofluid, emulsion, surfactant, etc.) or gas-based methods (injecting various gases such as carbon dioxide, hydrocarbon gas, nitrogen, flue gas, etc.) or a combination of these methods (injection of water alternating gas or injection of carbonated water, etc.). These methods have a major problem, especially gas-based methods. Which are the early breakthrough and the viscous fingering phenomenon. The reason for these problems is the low viscosity of these fluids^73–77^. In recent years, several methods have been considered to solve this problem and increase the efficiency of gas injection, especially in the case of carbon dioxide, including WAG injection, thickener injection, nanoparticles injection, and foam injection. In this section, the methods are divided into two categories: (1) methods based on gas-phase modifications, (2) other methods.

### Principals of gas-phase modification-based methods

One way to increase the gas phase viscosity is to use thickeners. These materials by increasing the viscosity of the gas, prevent the early gas breakthrough and viscous fingering, and increase the sweep efficiency. Gas thickening technology uses the dissolution of the “Viscosifier” compound to create a transparent, thermodynamically stable, rich, and high-pressure gas phase with increased viscosity^78^. So far, many studies have been performed to increase the solubility of thickeners to increase the viscosity of the gas phase. For this purpose, researchers are trying to balance gas-philic and gas-phobic groups. Using these thickeners significantly increases the efficiency of gas injection and can reduce the negative effects such as bypassed flow, and water blockage, can cause stability in the flow, and create the desired mobility ratio^79,80^. The thickening performance is such that it increases the viscosity by adding to the injection fluid and delays the breakthrough time by reducing the viscous fingering phenomenon^81,82^. It should also be noted that studies that have been conducted so far include problems such as toxicity of the materials used, uncertainty in the use of this technology, measurements method, high cost, and insufficient increase in viscosity.

#### Mechanism and technology of thickeners

Gas in supercritical conditions has unique properties such as low toxicity, low flammability, and, etc.^83^. Regarding gas injection, a few points should be noted: (1) the desired mobility cannot be achieved by pure gas at any pressure and temperature. (2) During gas injection, especially carbon dioxide, we are faced with viscous fingering phenomenon and early gas breakthrough time. (3) During gas injection, it is possible to face problems related to gas injection, especially carbon dioxide. Given these points, the need to use thickeners is felt more^84,85^. The presence of thickeners by dissolving in the gas increases its viscosity. In summary, this technology involves increasing the viscosity of the gas phase using special molecular structures that dissolve in the gas phase to create a transparent and stable phase at high pressure (thermodynamically). When the gas is enriched and the thickeners are dispersed in the gas phase, can provide the necessary force to overcome the capillary force that trapped the oil in micro pores and increases the production efficiency^86^.

As mentioned, the use of these materials can affect oil production during the gas-based EOR process. Materials that can be used as thickeners include polymers, small molecules, and surfactants. The effectiveness of these materials is different.

#### How to dissolve or dispersed solid particle in gas phase

The cloud point method is a common technique for evaluating the solubility/dispersivity of solid components in supercritical gas phase. In this method, should be used a HPHT visual cell. This system consists of mechanical stirrer, light source, high-pressure pump, thermal jacket, accumulator, and optical microscope. The cloud point method is particularly useful for the visual analysis of solid particles solubility/dispersivity. The process of this analysis includes several steps. First, the desired amount of solid material is placed inside the HPHT visual cell. After that, the system is vacuumed to ensure that no air remains in the system. After 30 min, if the pressure remains constant, the desired gas (CO2) enters the HPHT vision cell. The mechanical stirrer is then activated and the mixing process continues until the solid particles disappear in the gas phase (solid particles cannot be observed with a microscope). The system pressure is then reduced until the microscopic image is foggy. When the image of the magnet disappears completely, the recorded pressure is the cloud point pressure. Since this method is based on visual analysis, each analysis must be repeated three times to ensure the consistency and accuracy of the results.

#### Types of thickeners

**Figure 3:**
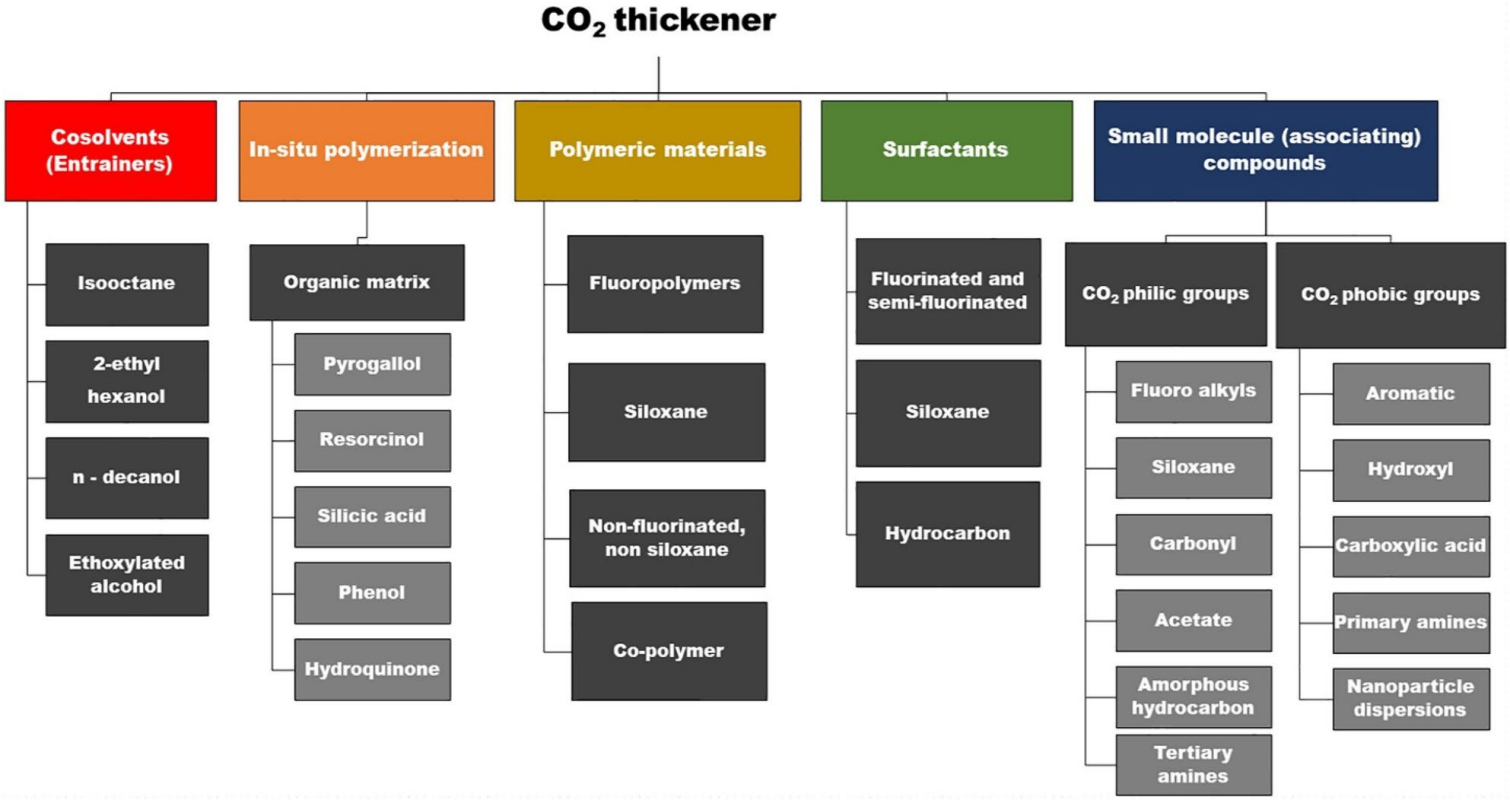
Types of thickeners^86^.

For a thickener to have a suitable performance and be able to perform the EOR process more efficiently, it must have a high solubility in the gas phase and increase the viscosity suitably. In general, thickeners are divided into two categories, direct and indirect, (depending on their function). These materials must be able to thicken the gas phase and increase viscosity. The thickening process is done by creating a hydration layer, aggregation, entanglement, self-assembly, and coil expansion. The classification of thickeners types is shown in the Fig. 3^87,88^.

#### Polymer-based CO2 thickener

Carbon dioxide thickening technology is divided into 4 categories: (1) polymeric thickeners, (2) small molecules, (3) surfactants, and (4) nanoparticles^89–91^. In this section, all methods will be discussed in detail along with the advantages and disadvantages of each method. Polymeric thickeners were first investigated in the 1970s^92–94^. Many attempts have been made to obtain polymeric thickeners, which have been reviewed in a comprehensive study by Enick et al. Studies have shown that polymers that have heteroatoms of silicon, nitrogen, oxygen, and halogens have a greater ability to thicken carbon dioxide^95–97^.The reason for this phenomenon is the more gas-philic properties of these compounds. Based on this, polymers that can thicken carbon dioxide are divided into four categories: (1) nitrogen-based, (2) silicon-based, (3) oxygen-based, (4) hydrocarbon-based, and (5) fluorinated-based^98–101^.

##### Fluorinated-based polymer

Fluorinated polymers are able to significantly dissolve in the gas phase without co-solvent due to their gas-philic properties. This property causes these materials to have a high ability to increase the viscosity of carbon dioxide. One of the biggest limitations of thickeners is their inability to dissolve significantly in the gas phase, which causes the viscosity of carbon dioxide not to change enough. So far, poly fluoro acrylates have been known as the best and most effective polymer in increasing the viscosity of carbon dioxide^102^. However, it should be pointed out that the environmental problems of this category of polymers are the reason for their limited use in field-scale EOR processes^99^. Enick et al., investigated the dissolution effect of styrene poly fluoro acrylate copolymer in carbon dioxide. They showed that this polymer is soluble in carbon dioxide, but its dissolution is inversely related to the concentration of styrene. Goicochea et al., stated that the efficiency of increasing the viscosity at low temperatures by polyFAST was much better than PFOA. Increasing the viscosity by two to three times is not ideal due to high costs and environmental impact^103^. In a similar study, Lemaire et al., investigated the effect of dissolving polyFAST in carbon dioxide on the viscosity of the gas phase. By adding 1.5%(w/w) of this polymer, the viscosity increased 19 times. This increase in viscosity occurred at a temperature of 25 °C and a pressure of 15 MPa^104^.

##### Silicon-based polymer

Siloxanes are another group of polymers that are very effective in increasing the viscosity of carbon dioxide. Silicon-oxygen bond is the main characteristic of these compounds. Polydimethylsiloxane is one of the popular thickeners for increasing the viscosity of carbon dioxide^105^. Zhao et al., investigated the effect of PDMS on increasing the viscosity of CO2. For this purpose, they used 5% (w/w) PDMS and 5% (w/w) co-solvent (kerosene). They showed that the viscosity of CO2 increased significantly (54 times compared to pure carbon dioxide). However, the field application of this material is not economical due to the high cost and the need for a high volume of co-solvent^106^.

##### Oxygen-based polymer

This type of polymers performed better than hydrocarbon polymers for CO2 thickening. In addition, they have more variety. This type of polymers consists of hydrogen and carbon atoms and oxygen heteroatoms. The presence of hydrogen heteroatoms in the structure of these materials makes these polymers perform better than hydrocarbon polymers. PVEE (poly(vinyl ethyl ether)) is one of the most famous polymers that can increase the viscosity of carbon dioxide up to 14 times^80^.

Another oxygenated polymer is polyvinyl acetate, which has shown promising results in increasing the CO2 viscosity. This polymer requires large volume co-solvent such as toluene to dissolve in CO2. This is one of the major limitations of using this polymer. Although it should be noted that this polymer is the second non-fluorinated polymer that is soluble in the gas phase after PDMS, and has been able to show good performance^107^.

One of the limitations of using polymers to increase the viscosity of the gas phase is the dissolution of these materials in CO2. Increasing the polymer concentration can significantly increase the dissolution pressure. In addition, the use of high concentrations of thickener may not be economical. Therefore, it is important to use polymers that can significantly increase the viscosity of the gas phase at low concentrations. Poly(benzoyl-vinyl acetate) (PolyBOVA) is one of the polymers that has been able to increase the viscosity of carbon dioxide even in low concentrations. However, its dissolution pressure in CO2 is very high^108^.

Zhang et al., studied the increase in CO2 viscosity due to the dissolution of poly(propylene oxide) (PPO). Polypropylene is a low molecular weight polymer that has a high solubility in CO2. The high dissolution of PPO in CO2 is due to the weak self-bonding of PPO-PPO and the strong interaction between CO2 and poly(propylene oxide). The study found that the increase in viscosity was only about 25%, which was not considered significant^109^. Many polymers have been used to increase the viscosity of CO2, including poly(propylene glycol) (ppg), poly(lactic acid) (PLA), poly(vinyl formate) (PVF), poly(Urethane disulfates (PUD)^103,105,110,111^.

##### Hydrocarbon-based polymer

This type of polymer has a lower ability to dissolve in CO2 due to the absence of oxygen, silicon, fluorine, and nitrogen heteroatoms in their structure. So far, the only hydrocarbon polymer that could significantly increase the viscosity of CO2 is poly(1-decene) (P-1-D). .Many attempts have been made to dissolve P-1-D in CO2 and increase its viscosity, which have also been unsuccessful. In 2022 Firozabadi investigated the performance of P-1-D in increasing the CO2 viscosity. All analyses were performed in reservoir conditions at 308 K and 31 MPa. They showed that the CO2 viscosity increased 6.5 times after dissolving P-1-D with a concentration of 1.8% (w/w)^90^. In 2023, Afra et al.^112^ showed that 1-decene oligomers can increase the CO2 viscosity by 4.8 times at a concentration of 1.5% (w/w) P-1-D, at 363 K and 24 MPa.

##### Nitrogen-based polymer

Nitrogen-based polymers are another group of thickeners that, despite the great variety of studies that have been conducted on them, could not show proper solubility in CO2 at low concentrations. This is the biggest limitation of this group of polymers in thickening the gas phase. The reason for this phenomenon is the strong polymer–polymer interaction compared to CO2-polymer interaction. For this reason, it is not recommended to use this type of polymers in laboratory studies^113^.

In general, in the use of polymers, parameters such as oil composition, formation water salinity, reservoir rock properties, need or lack of co-solvent, polymer concentration, the ability of the polymer to thicken CO2, solubility of the polymer in carbon dioxide should be considered. Polymer molecular weight and operational conditions such as temperature and pressure are also vital parameter. These parameters can significantly affect the conditions of use of thickeners in industrial scale. In addition to the mentioned parameters, environmental issues, the cost of polymers, the ability of polymers to increase the viscosity of the CO2 and mobility control are important issues for the commercialization of any polymer thickener.

##### Advantages and disadvantages

The application of polymers as carbon dioxide (CO2) thickeners has garnered significant attention recently. Numerous studies have been conducted to investigate the potential of polymers in this regard. However, each method has its advantages and disadvantages, which are discussed briefly. The active mechanism of carbon dioxide thickening by polymers is divided into four categories: (1) self-assembly, (2) entanglement, (3) intermolecular interactions, and (4) polymer coil expansion. The use of polymers is one of the methods that have been studied, and many of its uncertainties have been reduced. In addition, the performance of this method is better than other methods and could significantly increase the CO2 viscosity. This type of thickener has also been used in field scale and can significantly reduce mobility. In addition, polymers have the ability to efficiently thicken CO2 at different temperatures, from ambient temperature to reservoir temperature. It should be noted that many polymers are dissolved in carbon dioxide without co-solvent. However, the use of polymers has some limitations. Polymers with higher molecular weight can thicken more CO2. But these polymers have low solubility in CO2 and it is difficult to provide dissolution conditions. To use polymers with high molecular weight, co-solvents must be used, which are often expensive and not cost-effective. In addition, fluorinated polymers are not approved from the point of view of the environment, and they also have adsorbed on the surface, and this causes a limitation in the thickening of CO2. It should be noted that the polymer dissolution in CO2 mostly occurs in supercritical conditions, and this phenomenon causes in some cases the pressure required for the dissolution of the polymers higher than the MMP of carbon dioxide in oil.

#### Small self-interacting molecules

Another group of CO2 thickeners are self-interacting small molecules. This group of thickeners, like surfactants, has a gas-philic, and a gas-phobic group. The opposition of these two groups causes stability and dissolution of polymers in the gas phase^107^. In other words, the gas-philic group causes the polymer to dissolve in CO2. While, like other surfactants, the O2-phobic head can interact with other molecules. The power of the CO2-phobic head is very important. If the group associated with the thickener is strong, this material may be insoluble in the system. Conversely, if this group is weak, the donor can be soluble in the system. In general, as stated in the previous sections, for the proper performance of the thickener, the target material must be dissolved significantly in CO2 without the need for a high volume of co-solvent. These structures can create molecular bonds in the solution and increase the viscosity of the gas phase by forming macromolecules. Although it should be noted that at high temperatures, due to the reduction of intermolecular forces, the thickening property decreases significantly^104^.

##### Advantages and disadvantages

Small molecules can increase the viscosity of carbon dioxide and reduce mobility by creating macromolecular networks. Unlike polymers, these materials can also increase the viscosity of carbon dioxide in low concentrations. These molecules increase the viscosity of carbon dioxide by creating a gel in the fluid bulk. The system formed by small molecules is a single-phase and homogeneous viscous solution. But since these materials create a homogeneous and viscous phase, they need a co-solvent and heating to dissolve in CO2, which requires additional energy and cost for their dissolution. Due to their gel-like properties, their efficiency is reduced at high temperatures and they cannot increase viscosity like at low temperatures. When the concentration of thickeners is increased in CO2, it makes the flow of modified CO2 in a porous medium more challenging. These issues limit the practical use of this type of thickener on an industrial scale. To date, no reports have been made on pilot implementation of these thickeners on a field scale.

#### Surfactants

Surfactants are a group of chemicals that can significantly reduce the surface tension in a multiphase system due to having a hydrophilic head and a long hydrophobic chain. Based on their charge, surfactants are divided into four types: anionic, cationic, amphoteric and non-ionic. Surfactants are widely used in the oil and gas industry. One of the main applications of surfactants is to create stability in CO2-foam systems during the gas base EOR process^114,115^.

The use of surfactant to increase gas-based EOR efficiency is a conventional method to increase the viscosity of the gas phase and mobility control. As mentioned, mobility is a function of the relative permeability and viscosity of both phases. Surfactants can increase the efficiency of the gas-based EOR process by two mechanisms. Surfactant molecules can gather in worm-like structures and form a viscous system by being in a network structure. In addition, these materials can increase the viscosity of the aqueous solution by increasing the stability of the CO2 foam by forming micelles around the CO2 molecules. The second mechanism is related to the change of rock surface wettability and relative permeability control. As the wettability of the system changes to oil-phobic (water-wet or gas-wet), the CO2 foam has a greater tendency to spread over the surface and this can easily remove trapped oil in the system. In other words, the surface does not show any resistance to the oil flow, and the oil will experience minimal contact with the rock surface^116,117^.

So far, many studies have been done to increase the CO2 viscosity by surfactants. However, surfactants could not enough increase the viscosity of the CO2, and in limited studies, viscosity increase due to the dissolution of surfactants in carbon dioxide has been reported. The reason for this phenomenon is the low dissolution of surfactants in carbon dioxide. Commercial surfactants cannot be dissolved in CO2 and this causes the viscosity to not change much. To overcome this limitation, scientists tried to increase their dissolution in the gas phase by functionalizing surfactants. Surfactants that can dissolve in CO2 are divided into three general categories, oxygen-based, fluorine-based, and amine-based surfactants^118,119^.

##### Advantages and disadvantages

Surfactants have caused CO2 thickening and mobility control by using stable foam formation. This method has been used in field pilot studies due to its efficiency and proper mobility control and has shown promising results. By creating CO2 foam, surfactants reduce the relative permeability of CO2 in the porous medium and control viscous fingering. In addition, surfactants can significantly reduce the surface tension between oil and CO2 and cause the reduction of capillary forces. In addition, the CO2 foam can be used in the water-sensitive formation, or even in the drilling process could prevent mud loss. Surfactants, like fluorinated compounds, are adsorbed on the surface significantly. For this purpose, it has been suggested to increase the amount of surfactants, but it should be noted that with the increase the concentration of surfactants, the injection process may not be cost-effective. In addition, it should be noted that many surfactants lose their properties when exposed to saline water and high temperature, and this causes them to no longer have the desired efficiency.

#### Nanoparticles

The application of nanoparticles in cementing, drilling mud, and the ٍEOR process has been confirmed. The application of nanoparticles to thicken CO2 is limited to its use for foam stability. The nanoparticles have only been used on a laboratory scale. Graphene oxide, boron nitride, cobalt oxide, aluminum oxide, nickel oxide, titanium dioxide, iron oxide, copper oxide, silicon oxide, silver, and palladium nanoparticles have been studied so far to increase the stability of CO2 foam. One of the key features that contribute to the efficiency of nanoparticles to increase the stability of CO2 foam, is their uniform dispersion in the aqueous phase. Among the nanoparticles studied to date, silicon oxide nanoparticles have demonstrated a noteworthy ability to achieve uniform dispersion in the blue phase. In this context, al-Yousefi et al., conducted research on the utilization of modified silica nanoparticles as CO2 thickener. Their findings suggested that the use of modified silica nanoparticles can be a promising approach for CO2 thickening applications^120–122^.

One of the advantages of using nanoparticles with surfactants or instead of surfactants in the gas-based EOR process is the resistance of nanoparticles against the harsh conditions of the reservoir such as pressure and high temperature and relatively high salinity of formation water. Nanoparticles are more stable in harsh conditions compared to surfactants. They can increase the stability of CO2 foam in gas-based enhanced oil recovery (EOR) processes. The purpose of using nanoparticles is to decrease surface tension, alter wettability, and enhance the viscosity of the CO2^123^. The direct use of nanoparticles to increase the viscosity is very limited. Shah and Rashint investigated the effect of copper oxide nanoparticles on the thickening of CO2. They showed that by dispersing nanoparticles with a concentration of 1% (w/w) with PDMS as the co-solvent, could increase the viscosity of the CO2 by 140 times^124^.

Nanoparticles have shown promising results on the lab scale and have shown their ability to thicken carbon dioxide. However, all the studies have been in laboratory and simulation scales. The ability of nanoparticles as CO2 thickeners must be investigated at a larger scale. Laboratory studies are also limited and extensive laboratory, and simulation studies should be conducted to confirm the ability of nanoparticles as CO2 thickener.

##### Advantages and disadvantages

Studies related to nanoparticles are very limited and still need to be further investigated. By dispersing nanoparticles in aqueous solution, the stability of CO2-foam increases. These nanoparticles can increase the stability of the foam under harsh reservoir conditions and prevent the instability of the foam under high temperature. Nanoparticles are adsorbed on the rock surface due to their unique properties like surfactants and change rock wettability. In addition, they can reduce the surface tension. In this way, nanoparticles can increase the foam's ability to increase oil production. However, it should be noted that the direct use of nanoparticles has also been reported, but it was only limited to one study. For the direct use of nanoparticles as a CO2 thickener, the concentration of nanoparticles in the gas-phase must be high. It should be noted that nanoparticles have an optimal concentration, higher than that concentration, the nanoparticle-nanoparticle interaction will be stronger than nanoparticle-carbon dioxide interaction, and this phenomenon causes the accumulation of nanoparticles in the porous medium.

#### Carbon dioxide thickeners overview

**Figure 4:**
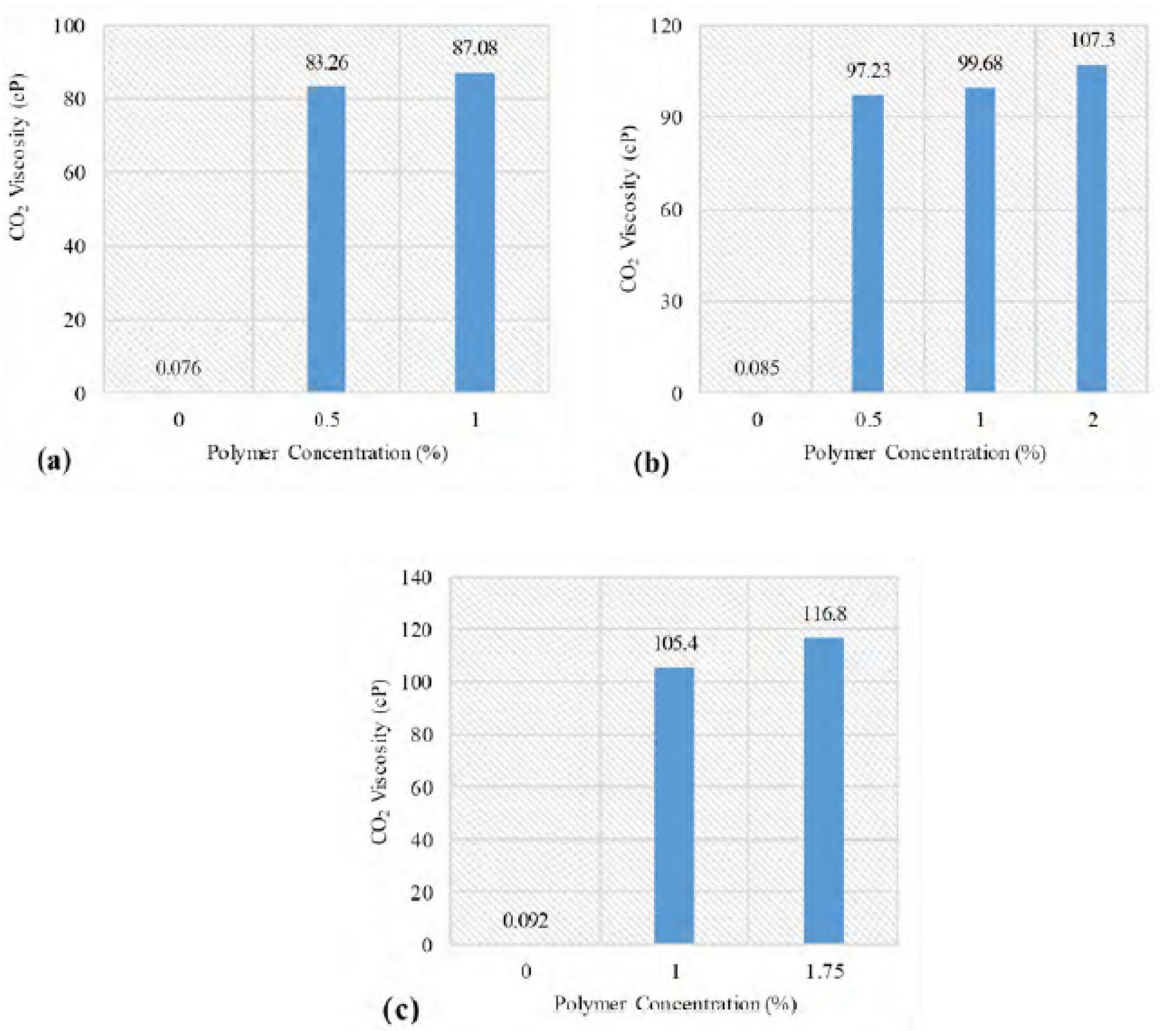
Effect of thickeners concentration and pressure (**a**) 1500. (**b**) 2000, (**c**) 2500) on viscosity of CO2^126^.

Heller et al. at 1985 studied the solubility of polymers in carbon dioxide to increase the gas phase viscosity. This study aimed to overcome the high mobility of the gas phase during carbon dioxide injection, which did not yield the desired results due to the slight increase in the viscosity of the modified gas phase. However, they stated that this study could lead to future studies and applications of polymers in increasing the gas phase viscosity so that gas-based methods can be considered instead of water-based methods to enhance oil recovery. Their results included the following: (1) a polymer was not found that was soluble in both water and gas phases. (2) The molecular weight of water-soluble polymers was higher than gas-soluble polymers (more than 6000). (3) The solubility of gas-soluble polymers is low, and this is one of the reasons why it does not achieve the appropriate viscosity. (4) Finally, they showed that water-soluble polymers are isotactic while gas-soluble polymers are amorphous and atactic^79^. In 2014, Lee et al., studied and designed carbon dioxide thickeners that contain small molecules. The structures the research team used to design the thickeners were non-fluorinated molecules. They stated that compounds containing the carboxylic acid agent were insoluble in carbon dioxide gas unless their gas-philic fraction increased. However, none of the samples tested in this experiment couldn’t increase the viscosity of CO2^125^. AlYousef et al., at 2019 investigated a new polymer because fluorinated polymers are environmentally hazardous. They stated that this material could be very effective and increase the viscosity of carbon dioxide from 100 to 1200 times. They showed that increasing the pressure would increase the viscosity of the gas in two ways: 1. Compression of the gas phase 2. Increase the solubility of thickeners in the gas phase. The results of this study can be seen in the Fig. 4^126^.

**Figure 5:**
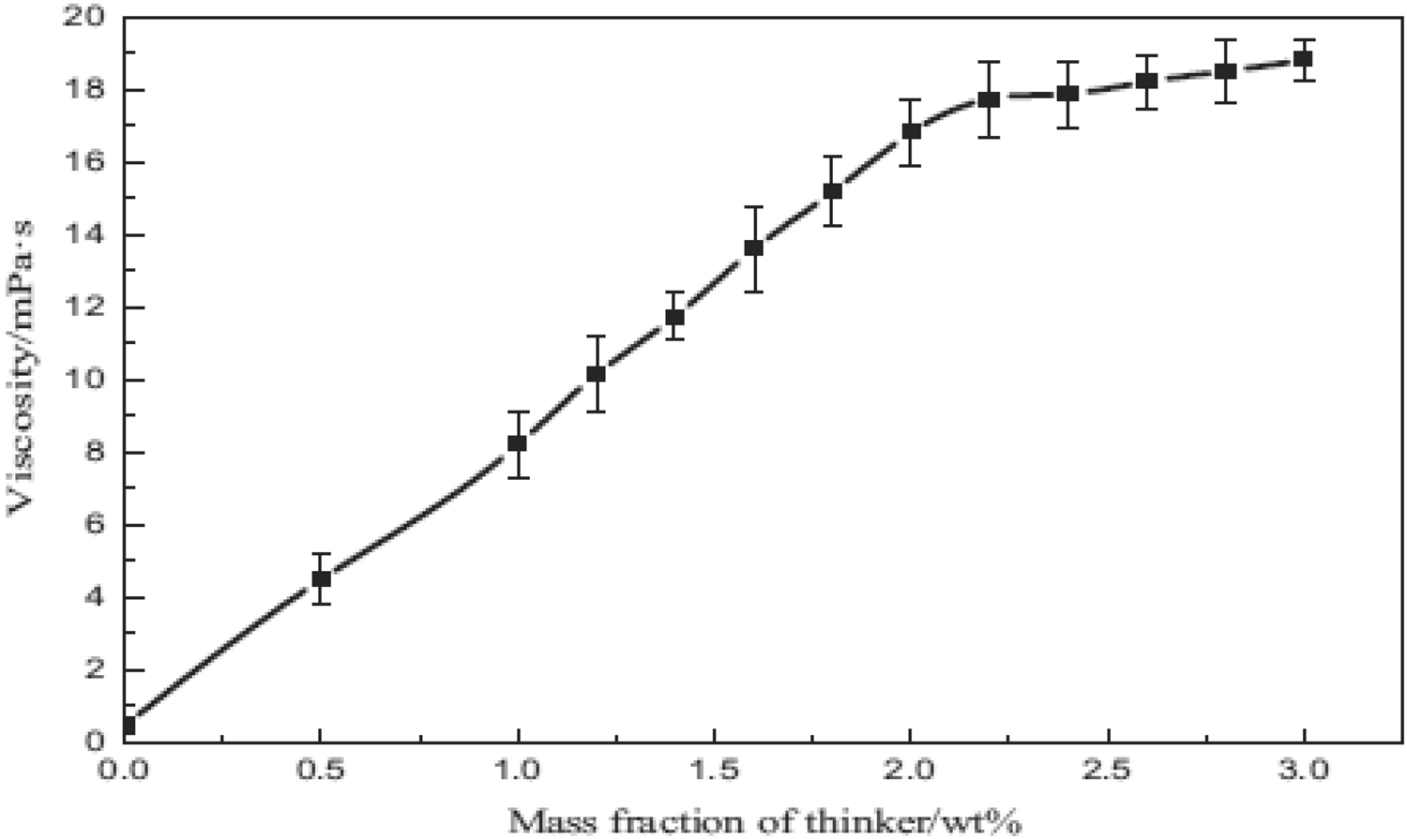
Effect of concentration of thickener on viscosity^129^.

Because some thickeners are not economically viable and are not environmentally safe, Al-Hinai et al., proposed a method to reduce their consumption. In this study, they examined two injection models, which are: (1) alternating injection of associated gas with thickener and associated gas without thickener (2) and alternating injection of carbon dioxide with thickener and associated gas. Their results showed that the alternating injection technique had similar results to the gas injection with only the thickener. They stated that by using this method, the consumption of thickeners could be drastically reduced^127^. Gandmakar et al., by focusing on carbon dioxide thickeners with small molecular sizes were trying to increase the gas phase viscosity to control the mobility ratio. They used a carbon dioxide-philic substance called PDMS. Their results showed that this material, in addition to increasing the viscosity of carbon dioxide by nearly 5 times, also reduced the minimum miscible pressure. They stated that according to these results, the use of this thickener can increase oil recovery by up to 15%^128^. In 2020, Zhou et al., studied an oligomeric thickener to increase the viscosity of carbon dioxide. They showed that the use of this substance could increase the viscosity of supercritical carbon dioxide by 480 times (this increase occurred at a concentration of 2% by weight of thickener). They stated that viscosity is directly related to thickener concentration and inversely related to temperature and shear stress. The main reason for the efficiency of this material in increasing the viscosity of the gas phase is due to the unique structure of this material, which includes two groups of urea and four groups of fluorocarbons (Fig. 5)^129^.

Zhang et al., to control the gas phase mobility ratio, examined two commercial thickeners to increase the viscosity of carbon dioxide. They used P-1-D and PVEE in this study and showed that the minimum dissolution pressure of PVEE was much lower than P-1-D. They also stated that the use of these polymers could improve oil recovery and drastically delay gas breakthrough time. This is due to the increase in gas phase viscosity (approximately 14 times higher than the pure state) and the decrease in surface tension between the oil and the polymer-modified gas phase^80^.

The solubility of surfactants in the gas phase is one of the parameters that has attracted much attention since the 1990s, but not many observations have been made regarding the increase in the viscosity of the gas phase during the dissolution of the surfactants^130,131^. Consan et al., conducted extensive studies on the dissolution of surfactants in the gas phase. In this study, they examined more than 130 types of surfactants. Their results showed that among all these surfactants, non-ionic surfactants have the potential for solubility in the gas phase^132^. Hoefling et al., decided to synthesize fluorinated surfactants because these compounds have a high ability to dissolve in the gas phase and are inherently gas-philic. Their results showed that these surfactants can be dissolved in the gas phase at moderate pressures^133^. In the continuation of these studies, Harrison et al. Synthesized the surfactant C7F15CH (OSO3Na+) C7H15. Their results showed that this combined surfactant could create a water-in-gas emulsion^134^. Zhao et al., also investigated the solubility of the surfactant in the gas phase and related parameters. They stated that the structure of the surfactant plays an important role in its solubility in the gas phase, so their results showed that branched alkyls will have more solubility in the gas phase than linear alkyls. Nonlinear and branched structures prevent the deposition of surfactants due to their greater strength and the proximity of surfactant molecules. Nonlinear and branched structures prevent the deposition of surfactants due to their greater strength and prevention of surfactant aggregation. As mentioned, none of the studies mentioned above reported an increase in viscosity when using the surfactant^81^.

Self-assembly surfactants have the potential to increase the gas phase viscosity. The mechanism of viscosity increase in these surfactants is such that the micelles engage with each other in an optimal concentration and form a network structure that increases the gas phase viscosity. Changing the state of micelles to a rods-like shape can be one of the mechanisms to increase viscosity^135^. Trickett et al., investigated the same issue. They added Ni (di-HCF4) 2 at 10% (w/w) and observed that the viscosity of carbon dioxide increased significantly (90%). However, the spherical micelles formed by Na (di-HCF4) did not perform properly, and the viscosity did not change much^135^. Sagisaka et al., investigated the effect of anionic hybrid surfactants on the viscosity of carbon dioxide. They stated that temperature and pressure are very important parameters in changing the viscosity of the gas phase. They showed that by changing the temperature and pressure, the viscosity could be increased by up to 200%^136^. According to the results obtained from these researchers, it can be said that by designing suitable structures, the viscosity of carbon dioxide can be greatly increased by surfactants.

The Table 2 summarizes the studies on carbon dioxide thickeners.

**Table 2:** A summary of studies on CO2 thickeners.

Author	Material	Thickener type	Results	References
Llave et al.	Isooctane Ethoxylated alcohols 2-Ethyl hexanol n-Decanol	entrainers	Increasing the solubility of oil components in carbon dioxide and increasing the viscosity, but due to the high volume of materials used, this method was not cost-effective	^137^
Terry et al.	polymerization of Ethylene, octane, decane	in situ polymerization	Increased solubility in carbon dioxide	^138^
Lancaster et al.	Organic titanate with silicic acid pyrogallol, hydroquinone, resorcinol, phenol	in situ polymerization	Insolubility in liquid carbon dioxide and no increase in viscosity due to deposition	^139^
Heller and Dandge	53 polymers were tested, of which 17 cases were soluble in carbon dioxide	Polymer materials	Their results showed that the highest solubility was related to poly (1-decene)	^79^
Shi et al. Enick et al. Xu et al.	Fluorinated compounds with a molecular weight of 13,000–30,000 g/mol	Fluoropolymer	These compounds showed good solubility in the gas phase. But they could not change the viscosity significantly	^140–142^
Combes et al. Guan et al.	Fluorinated acrylate Homopolymer	Fluoropolymer	In this work, by adding 5–10% of this material, the viscosity of carbon dioxide can be increased 3 to 6 times	^143,144^
Shen et al.	poly(fluoroalkyl acrylate) (PFA)	Fluoropolymer	This material showed higher solubility than poly (vinyl acetate)	^145^
Fink et al.	modified siloxane oligomer	Siloxane polymers	They were able to increase the viscosity and solubility of this material in carbon dioxide (by functionalization of siloxane)	^146^
Bac and Irani	polysiloxanes	Siloxane polymers	They showed that the viscosity increased significantly (90 times), the production rate increased, and delay in breakthrough time	^147^
Williams et al.	poly (dimethylsiloxane)	Siloxane polymers	They showed that the viscosity increased appropriately. And the dissolution pressure decreased with the addition of co-solvent	^148^
Doherty et al.	propyltris(trimethylsiloxy)	Siloxane polymers	They showed that the material could increase viscosity by up to 300 times, depending on the type of co-solvent used	^149^
Rousseau et al.	poly (dimethyl siloxane)	Siloxane polymers	This material has shown high solubility	^150^
Sun et al.	PDMS methyl-benzene	Siloxane polymers	They showed that this material can increase the viscosity to a desirable level	^151^
Conway et al.	polylactides	Other polymers	The solubility of these materials is high in carbon dioxide	^152^
McClain et al.	poly(1,1-dihydro-perfluorooctylacrylate)	Co-polymer	They showed that this substance has good solubility in carbon dioxide, but to achieve high viscosity requires a high concentration of this substance	^153^
Sarbu et al.	poly(ether carbonate)	Co-polymer	They showed that this material could be dissolved in carbon dioxide at low pressures	^154^
Huang et al.	polyFAST	Co-polymer	The results showed that this substance was able to change the viscosity of the gas phase from 5 to 400 times in different concentrations, and gas injection tests to the core also showed a 19-fold increase in viscosity	^155^
Xu et al.	polyFAST	Co-polymer	They stated that this material has a high solubility in the gas phase due to the presence of Fluoroacrylate, but cannot be used in oil fields due to high costs and environmental problems	^142^
Kilic et al.	styrene-HFDA	Co-polymer	They showed that styrene-HFDA was better than poly-HFDA in increasing viscosity	^156^
Heitz et al.	Ammonium carboxylate perfluoropolyether	Tailor-made surfactants	The solubility of this substance in carbon dioxide was significant	^157^
Psathas et al.	Copolymers obtained from poly (methylacrylate) and poly (dimethylsiloxane)	Tailor-made surfactants	The results showed that the use of these materials improves viscosity	^158^
Da Rocha et al.	Surfactant based on trisiloxane	Tailor-made surfactants	They stated that fluoride and siloxane-based surfactants are not cost-effective and cause environmental problems	^130^
Doherty et al. Shi et al.	fluorinated compounds with urea	Small molecules	All of these materials have shown good solubility in carbon dioxide but do not necessarily increase viscosity	^149,159^
Gullapalli et al.	12-hydroxystearic acid with co-solvent	Small molecules	This compound can increase the viscosity of carbon dioxide by 58%	^160^
Afra et al.	P-1-D	Co-polymer	The use of this thickener increased the carbon dioxide storage capacity	^112^
Zhang et al.	PPO	Co-polymer	This polymer was significantly absorbed in carbon dioxide and increased the viscosity of carbon dioxide by 1.25 times	^109^
Zhao et al.	PDMS	Co-polymer	They showed that polymer dissolution in carbon dioxide occurs at lower pressures by using co-solvents	^106^
Lemaire et al.	PDMS PVAc	Co-polymer	They investigated different thickeners and their co-solvents. In this study, the effectiveness of each of the thickeners has been investigated	^104^
Gandomkar et al.	PFA	Co-polymer	They found that after carbon dioxide modification by using polymer, the amount of oil production increased by 16%	^161^
Tadepalli et al.	PolyFast	Co-polymer	The results of molecular dynamics simulations showed that PDMS could not increase the viscosity of carbon dioxide, but PolyFast was able to increase the viscosity significantly	^91^

##### Hydrocarbon gas thickeners

Most studies in the literature are about the suitable thickener for carbon dioxide, but in the late 1969s studies were conducted about the hydrocarbon gases thickeners. The first attempt to provide a suitable thickener for hydrocarbon gas was performed by Henderson et al. In this experiment, they used three polymers of polyalkyl styrene, polymethyl lauryl, and polybutadiene to thicken the gas phase. The results showed that the viscosity of the gas phase could increase to about 0.1%, which occurs at a concentration of 0.25% (v/v)^162^. Dauben et al., continued studies to increase the viscosity of hydrocarbon gases. They were able to increase the viscosity of condensate (75% propane and 25% heptane) up to 5 times by using polyisobutylene polymer. (This increase in viscosity occurs at a concentration of 0.25% (w/w) of PIB)^163^. Dandge and Heller used (PAOs) polymers to increase the viscosity of LPG and carbon dioxide. In this study, they showed that these polymers dissolve relatively well at a certain temperature and pressure in hydrocarbon gas, but their dissolution in carbon dioxide gas will be more limited than in hydrocarbon gas. They stated that when the concentration of these polymers increases by 2.2% (w/w), it can increase the viscosity of hydrocarbon gas (butane) up to five times^164^. Dhuwe et al., also studied three types of polymers (DRA poly-α-olefin, PDMS and PIB) to study the suitable thickener for hydrocarbon gas. They stated that by adding hexane to the system, the solubility of DRA polymer in the gas phase increased, and as a result, an increase in viscosity will be observed. Their results showed that at a concentration of 0.5% (w/w) of this polymer (at the presence of hexane at a concentration of about 24% (w/w)) the viscosity of ethane and propane increased up to 9 times, and the viscosity of butane up to 32 times. The reason for the considerable difference is the solubility of the polymer in different gases. They also stated about the other polymers that PIB is completely insoluble in the hydrocarbon gas, and PDMS is soluble in the gas phase without any solvent^165,166^.

**Figure 6:**
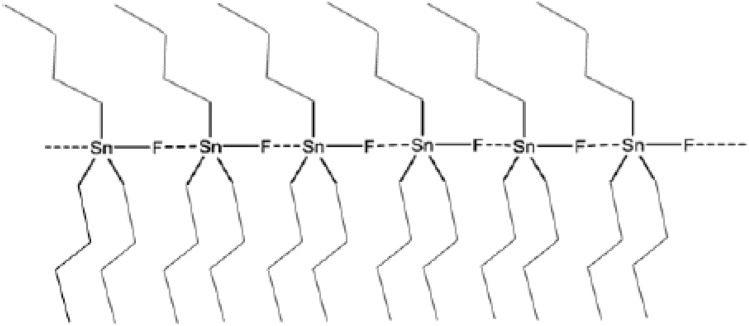
Structure and mechanism of connection of TBTF components^167^.

Don and Oldfield used TBTF as a thickener for hydrocarbon and non-polar gases for the first time. This material is a white powder whose structure and mechanism of connection of its components can be seen in the Fig. 6. The results show that this material can be easily dissolved in organic gases and does not require any other solvent or heating. Their observations of the gas phase viscosity behavior (moderate hydrocarbons) after modification showed that this material could be used as a thickener^167^. Further studies on TBTF continued by Dandge et al., they showed that this substance is well dissolved in propane and butane and can increase viscosity up to 10 times, but its solubility in ethane is very limited, and it is not able to increase the viscosity of the gas phase^168^. Enick et al., showed that the substance would dissolve up to 5% (w/w) in hydrocarbon gases such as butane and propane^169^. Among all the compounds, in this case, TBTF has so far shown the best performance in increasing viscosity at a certain concentration. For example, this material was able to increase the viscosity of normal hexane up to 750 times, but in the case of tetrachloroethylene, this increase is almost halved (380 times)^168^.

HAD2EH is another substance that can increase the viscosity of hydrocarbon gases. Enick et al., examined HAD2EH as a thickener for hydrocarbon gases and found that it could significantly increase the viscosity of light hydrocarbons (butane and propane) due to its good solubility in the gas phase. The results showed that by increasing the concentration of this material up to 1% (w/w), the viscosity of the gas increases up to 100 times. As shown in prior studies, HAD2EH is insoluble in light hydrocarbons such as ethane and completely soluble in moderate hydrocarbons such as propane and butane. These results were completely agreeing with the observations of Dhuwe et al. In this work, they studied the behavior of HAD2EH with different temperatures, pressures, and different gases. They showed that HAD2EH could increase the viscosity of butane and propane by 19 and 3 times, respectively, however, it doesn’t change the viscosity of ethane because HAD2EH does not dissolve in the light ga.

Nanocomposites have also been able to play a role in increasing the viscosity of hydrocarbon gases as new material. In 2020, Gandmakar and Sharifi were able to dramatically increase the viscosity of the hydrocarbon gas phase by using a new nanocomposite. In this study, they used P-1-D and its nanocomposites with graphene oxide. The results showed that this nanocomposite was able to reduce the minimum miscible pressure without co-solvent. The results show that the gas phase viscosity increased 4.5 times using P-1-D and 22.9 times using nanocomposite. They stated that the use of nanocomposites has drastically reduced the interfacial tension, and in addition to improving mobility, this has increased production and reduced residual oil by up to 24%^170^.

### Water alternating gas injection

**Figure 7:**
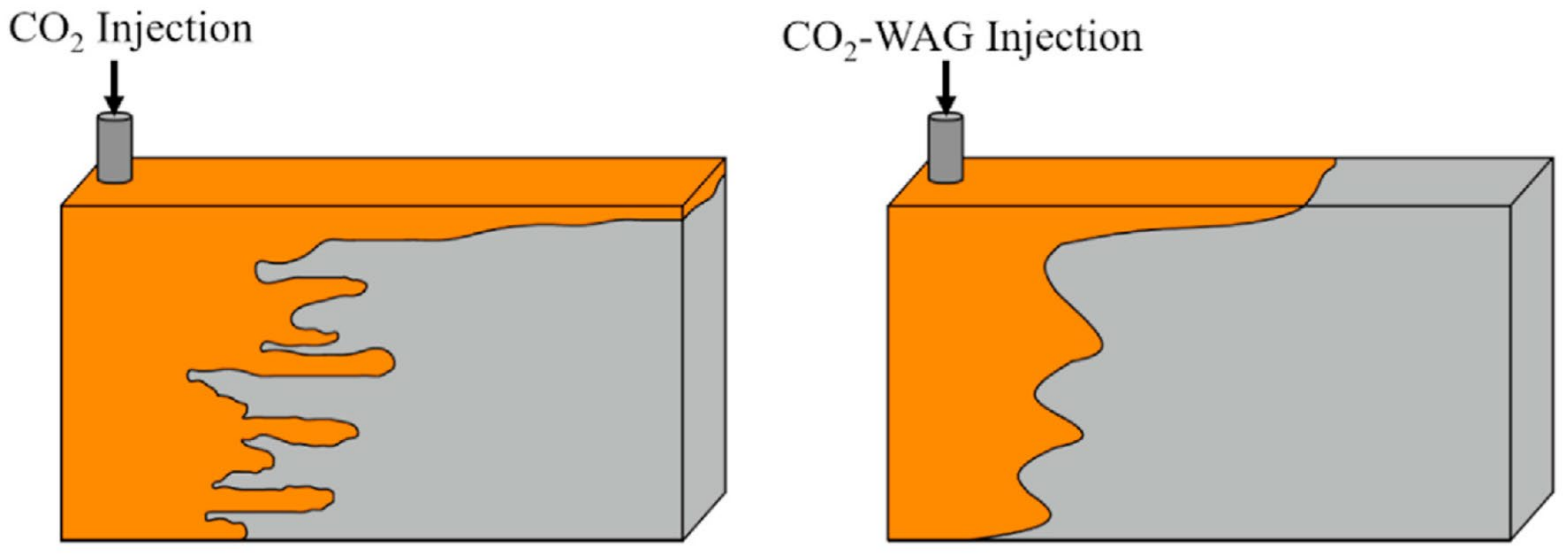
Effect of water alternating gas injection on improving the mobility ratio^184^.

Water alternating gas injection is one of the methods used in oil industry to overcome undesirable mobility ratio. This method has the characteristics of both water injection methods (high macroscopic efficiency) and gas injection (high microscopic efficiency). Using this method reduces the limitations of gas injection and water injection^171–175^. This method is very effective in layered and high heterogeneity reservoirs because it reduces the movement of gas in the permeable layers and makes the fluid front more homogeneous. It should be noted that this method consumes less gas than continuous gas injection^176–178^. Go and Han have done some research on this field. In this research, they used carbon dioxide as the gas phase. They stated that injection of water alternating gas due to the improvement of macroscopic sweep efficiency (presence of water), and microscopic sweep efficiency (presence of carbon dioxide), can significantly increase production by controlling the mobility ratio^179^. Dai et al., also examined the benefits of water alternating gas injection. They stated that to maximize production in this method, we should obtain parameters such as water to gas injection ratio, injection phase time, and distance between injection and production wells based on reservoir parameters^180^. Many researchers have used this method to control the mobility ratio, and many field applications of this method to delay gas breakthrough have been investigated in the literature^23,95,181–184^. As mentioned, the water alternating gas injection method can be used to delay gas breakthrough and control the gas mobility ratio in field applications. This process controls the mobility ratio in both miscible and immiscible conditions. The mechanism that reduces the gas mobility ratio is the reduction of gas-phase relative permeability due to the increase of water saturation in the porous media. To have a successful intermittent water and gas injection process and get the maximum amount of production, it is better to check all the reservoir parameters so that we can optimize the injection parameters. WAG injection can minimize the viscous fingering, delay early gas breakthrough, and increase the yield of the EOR process, but it cannot prevent gravity override (Fig. 7)^185^.

### Mobility control field studies

In this section, field-scale studies to control the mobility ratio are investigated. Most field reports have been about the use of water alternating gas injection or foam injection, and other methods have received less attention.

#### WAG injection

In 1988, Hsie and Moore investigated the effect of the water alternating and gas injection on the yield of EOR process from the Quarantine Bay sandstone reservoir. They showed that due to the miscible WAG injection, the recovery efficiency of this reservoir has increased by almost 17%, which shows promising results for this method on the field scale^185^. Kumar and Eibeck conducted a similar study on the WAG injection in a pilot of the sandstone formation. Their results showed that miscible WAG injection could increase the yield of recovery by 11%, which indicates the success of this process in sandstone reservoirs^186^. Bou-Mikael examined the process of WAG injection in a sandstone reservoir. This reservoir is located in Texas and has high porosity and permeability. The results of this study showed that this method could improve oil recovery and delayed gas breakthrough^187^. Brokmeyer et al., investigated the performance of WAG injection in the Lost Soldier Tensleep sandstone field. The field is located in Wyoming with a permeability of 31 mD and porosity of nearly 10%^188^.

#### Polymer thickeners

As mentioned in the previous section, direct CO2 thickeners are an almost new technology^189^. The conducted studies in this field are mostly on simulation and laboratory scale, and limited industrial applications of these cases have been reported. Most field studies have focused on polymers and surfactants as thickeners, and there is no pilot study for nanoparticles or small molecules. One of the successful pilots of carbon dioxide thickening has been reported in a field in northwestern Colorado. In this pilot, to thicken carbon dioxide, polyacrylamide is used. In this field study, 44 wells were injected with carbon dioxide thickened by polyacrylamide. They stated that after the injection of thickened carbon dioxide, the mobility of carbon dioxide decreased significantly, and this caused the oil production to increase by about 20 bbl/d^190^. In a similar field study, Karaoguz et al., investigated the effect of carbon dioxide thickening on the gas-based EOR process in a heavy oil reservoir in Turkey. They injected polyacrylamide into three wells and demonstrated a daily increase in oil production of 438 bbl/d, which is technically and economically feasible^191^. In an oil field in the USA, Muniz and Lantz conducted a field study of semi-hydrolyzed polyacrylamide to improve the gas-based EOR process. They stated that with the thickening of the CO2, the gas-oil ratio has decreased drastically and oil production has increased by 90,000 barrels in 1.5 years. Another method used in the field to conformance control of carbon dioxide is the use of gel polymers^191^. In this regard, Albidrez et al., used a crystalline superabsorbent copolymer (CSC). It swells on contact with water and has a resistance to degradation by carbon dioxide. The results obtained from injection and recovery diagrams show the positive effect of this material on the field scale^192^.

#### Surfactants

Surfactants are another group of thickeners that can thicken carbon dioxide on the field scale. For the first time, the use of carbon dioxide foam in the Rock Creek field in the United States of America was reported by Heller et al.^193^. They used Alipal CD-128 surfactant to create foam. They showed that carbon dioxide conformance problems were significantly reduced by using CO2 foam. The Rangely Weber Sand field in the USA saw an increase in oil production and carbon dioxide mobility control through the use of surfactants that are soluble in carbon dioxide, as reported by Jonas et al.^194^. They stated that by injecting CO2 foam into the reservoir, oil production has increased significantly, and viscous fingering has been controlled. In a similar study, Chou et al., showed that the use of surfactants in carbon dioxide injection could increase oil production in the North Ward-Estes field in the USA^194^. Martin et al. showed that CO2 foam could reduce the mobility of Nesselt to pure carbon dioxide by 66%^195^. Hoffner and Evans have also shown that CO2 foam could increase the efficiency of the EOR process in four test wells in two different states of USA by 10–30%. They stated that the CO2 mobility has increased significantly. Sanders et al., by using carbon dioxide foam, could increase oil production by 30% and reduce mobility by 50% in the SACROC field in West Texas. Alcorn et al. examined a foam injection in a pilot in the East field of Seminole Square. This field has an average permeability of 13 mD, but it has high permeable fractures that cause poor performance in the gas-based EOR process. The results showed that the use of ethoxylated alcohol could improve the mobility ratio of carbon dioxide^196^. Mirzaei et al., used a soluble carbon dioxide surfactant (ELEVATE ™) to make foam. The results showed that the problems related to conformance were reduced, but due to the lack of proper simulation, they couldn’t analyze the results^197^. Mukherjee et al., studied gas injection in a field located in the USA with a porosity of 14% and a permeability of 42 mD. The results of the gas injection were promising and the yield of recovery was suitable, but the gas to liquid ratio was increasing. To solve this problem, they did a foam injection pilot. The results showed that the problem was related to phase separation, the high gas to liquid ratio was improved, and the pilot test was successful^198^. According to the field scale studies, the results obtained from polymer and surfactant thickeners are promising and could significantly increase the gas-based EOR process’s efficiency. However, finding a stable thickener in reservoir conditions that can significantly increase the viscosity of the injected phase, and is suitable from the environmental and economic point of view requires extensive studies.

## Inhibition of asphaltene precipitation

Asphaltenes are known as the heaviest and polar components of crude oil, which is not soluble in light hydrocarbons such as normal pentane or normal heptane but is soluble in aromatic hydrocarbons such as toluene^199,200^. Asphaltene molecules have both aliphatic and aromatic structures. The existence of complex chemical and physical structures and properties is one of the challenges facing researchers^201–204^. In general, asphaltene particles in oil tend to coalesce to form larger particles, which is the beginning of the industry's challenges in asphaltene deposition^205,206^. Changes in the thermodynamic conditions of the oil by changing the temperature and pressure (during production from reservoir to separator) or changes in the composition of the oil (during EOR processes or gas storage) cause thinning resin layer around the asphaltene and precipitation of asphaltene. Removal and preventing this damage in the formation is one of the regular challenges of oil engineers^207–209^. One of the methods that are always used in removing or preventing asphaltene deposition is the injection of chemicals^210,211^. These chemicals include coagulants, anticoagulants, and non-polar solvents^212^. Use of these methods along with the appropriate initial efficiency has many disadvantages that make it necessary to use an alternative method. Disadvantages of these methods include (1) high volume of fluid consumption, (2) temporary and low durability, (3) need for more equipment and labor, (4) changing oil composition, (5) increasing operating costs, (6) uncertainty of asphaltene re-deposition. Given the above, providing and using materials that minimize these problems and have good efficiencies in preventing asphaltene deposition has always been challenging. In recent years, the use of nanotechnology in petroleum engineering has received much attention^213–216^. Nanoparticles due to their unique properties (high surface to volume ratio) can absorb heavy compounds such as asphaltene and prevent its deposition and delay this process^217–220^. In addition, nanoparticles can increase oil production and play an important role in EOR processes by reducing the interfacial tension between the injection phase and the oil in the reservoir. Nanoparticles are materials that have dimensions between 1 and 100 nm. These materials can be used in petroleum processes and in porous media (except nano-sheet or nano-tube) without pore blockage^221–223^. Studies in recent years have shown that nanoparticles such as SiO2, Fe3O4, Fe2O3, ZrO2, TiO2, and Al2O3 can reduce the hydrodynamic ratio of asphaltene particles to prevent asphaltene aggregation and deposition^224–227^. Nanoparticles can remove and adsorb asphaltene from crude oil due to their good dispersion in the oil phase, chemical reactivity, and high active surface area. In addition, nanoparticles can play an important role in EOR processes by reducing interfacial tension and changing the wettability of reservoir rock^228–231^. Nanoparticles can improve oil recovery through mechanisms that include: (1) emulsion formation, (2) improvement of heat capacity, (3) improvement of injection fluid density, (4) improvement of rock-fluid interaction, (5) improve rheological properties (viscosity). By combining nanoparticles with surfactants, polymers, or even other nanoparticles, the efficiency of the EOR process can be improved^232–238^.

### The effect of nanoparticles on the water-based EOR process

According to section two, common methods of asphaltene deposition treatment include surfactant injection, mechanical operations, and solvent injection^239,240^. These methods in addition to requiring complex equipment and requiring more labor, are very expensive (have a high operating cost) and don’t prevent asphaltene re-deposition. According to the reasons mentioned above, researchers are always looking for alternatives that, in addition to being highly efficient, are environmentally safe, prevent asphaltene re-deposition, and are cost-effective^241,242^. One of the fields that have received a lot of attention in recent years is the use of nanotechnology to absorb asphaltene and prevent its deposition. Nanoparticles have a high ability to prevent the aggregation of asphaltene particles, and deposition due to their high suspension rate, high adsorption, and high surface to volume ratio. The results of experimental studies by many researchers support this claim^243,244^. For example, Mohammadi et al., investigated the process of asphaltene adsorption on SiO2, TiO2, and ZrO2 nanoparticles. They showed that titanium oxide nanoparticles had the best performance due to the creation of hydrogen bonds in acidic conditions while no bonds were observed under basic conditions. Kazemzadeh et al., investigated the adsorption of three nanoparticles of iron oxide, silicon oxide, and nickel oxide on a glass module. The results showed that the highest adsorption was related to silicon nanoparticles and the lowest adsorption among these three nanoparticles was iron oxide^245^. Franco et al., examined different nanoparticles and their effect on asphaltene deposition in their study. In this experiment, they used 12 different nanoparticles at reservoir temperature, and pressure. Flooding of the oil-saturated core by normal heptane caused formation damage. The results showed that with the injection of nanoparticles with normal heptane compared to the injection of pure normal heptane, the relative permeability increased sharply, which indicates a significant reduction in the formation damage during the use of nanoparticles. They stated that the nanoparticles were able to prevent asphaltene precipitation and formation damage. In addition, oil production also increased^237^. Betancur et al., investigated the effect of size and acidity of nanoparticles on asphaltene adsorption. To do this, they used nanoparticles with dimensions of 11–240 nm. They stated that the best performance among nanoparticles belongs to nanoparticles that have higher surface acidity, smaller particle size, and higher adsorption capacity. They showed that treatment with nanofluids containing silicon oxide could increase oil production by 11% and improve the relative permeability of oil^210^. In a field study, Zabala et al., investigated the effect of injecting alumina-containing nanofluids into an oil field to prevent asphaltene deposition. The experiments were initially performed on the core scale, and after obtaining promising results, the nanofluid was injected into an oil field in Colombia. The results were very promising and oil production increased. They stated that the alumina nanofluid remained in the reservoir for eight months and could be used in the low permeability reservoir to prevent asphaltene deposition^246^. In another study, Taborda et al., investigated the performance of nanoparticles of aluminum oxide, acidic silicon oxide, and silicon oxide on asphaltene deposition in synthetic oil samples. The results showed that the best performance was related to aluminum oxide nanoparticles and the weakest performance was related to acidic silica nanoparticles, but the authors stated that all three nanoparticles were able to prevent the aggregation and deposition of asphaltene particles^242^. In a similar study, Shojaati et al., evaluated the effect of three nanoparticles, Fe3O4, NiO, and Al2O3, on delaying the onset of asphaltene precipitation. The results were very promising and showed that all of these nanoparticles have the potential to prevent asphaltene precipitation^243^. Lopez et al. studied the behavior of asphaltene in a synthetic oil sample by using nanocomposites. They showed that Cardanol/SiO2 reduces the size of asphaltene particles and prevents their aggregation due to its high ability to adsorb asphaltene particles in the sample bulk. They stated that this nanocomposite would have great potential to inhibit asphaltene precipitation^244^.

### The effect of nanoparticles on gas base EOR processes

#### Nanoparticles dissolved in the Liquid phase

**Figure 8:**
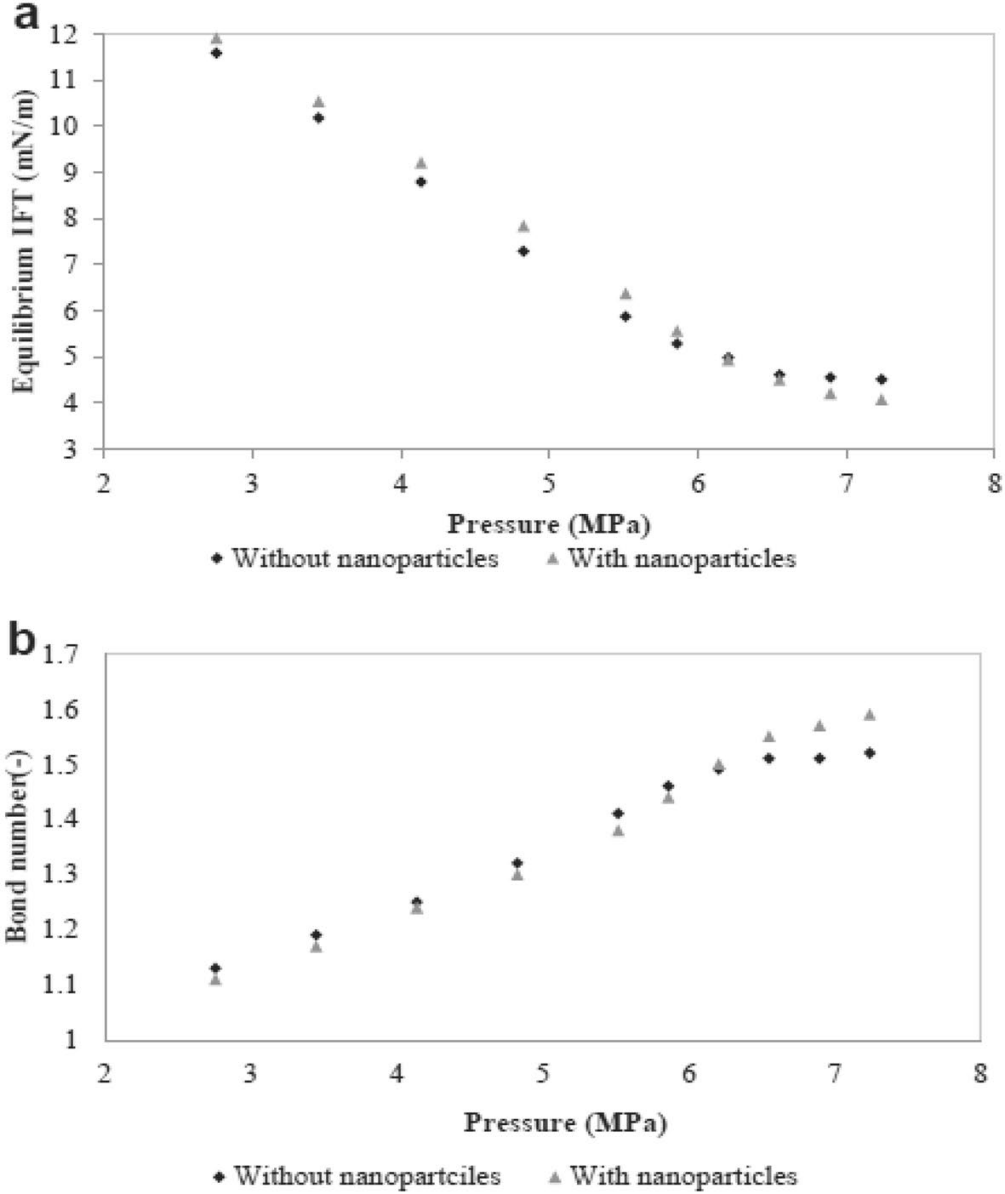
Effect of nanoparticle on IFT and bond number^245^.

Kazemzadeh et al., studied the active mechanism behind the impact of nanoparticles on asphaltene deposition during gas injection. In this study, they used interfacial tension and bond number to investigate the onset of asphaltene deposition. The nanoparticle used in this study was iron oxide and they used two models of synthetic oil with a combination of asphaltene, toluene, and normal heptane. Their results showed that with increasing the weight percentage of nanoparticles in synthetic oil, the intensity of asphaltene precipitation will decrease significantly. They also stated that the structure of asphaltene is one of the parameters that strongly affect the adsorption process by nanoparticles and the structure of asphaltene should be investigated to observe the best performance of nanoparticles (Fig. 8)^245^.

**Figure 9:**
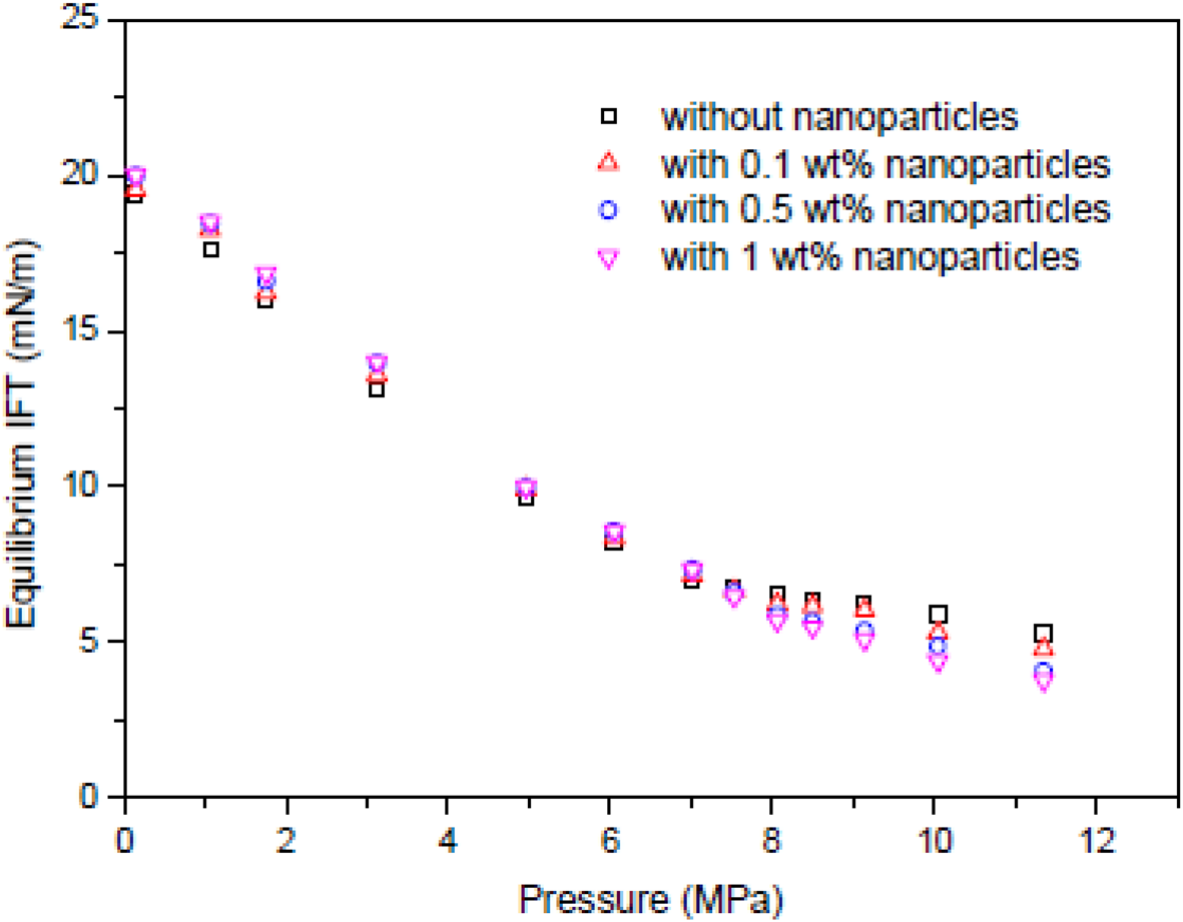
Effect of nanoparticle concentration on IFT^248^.

**Figure 10:**
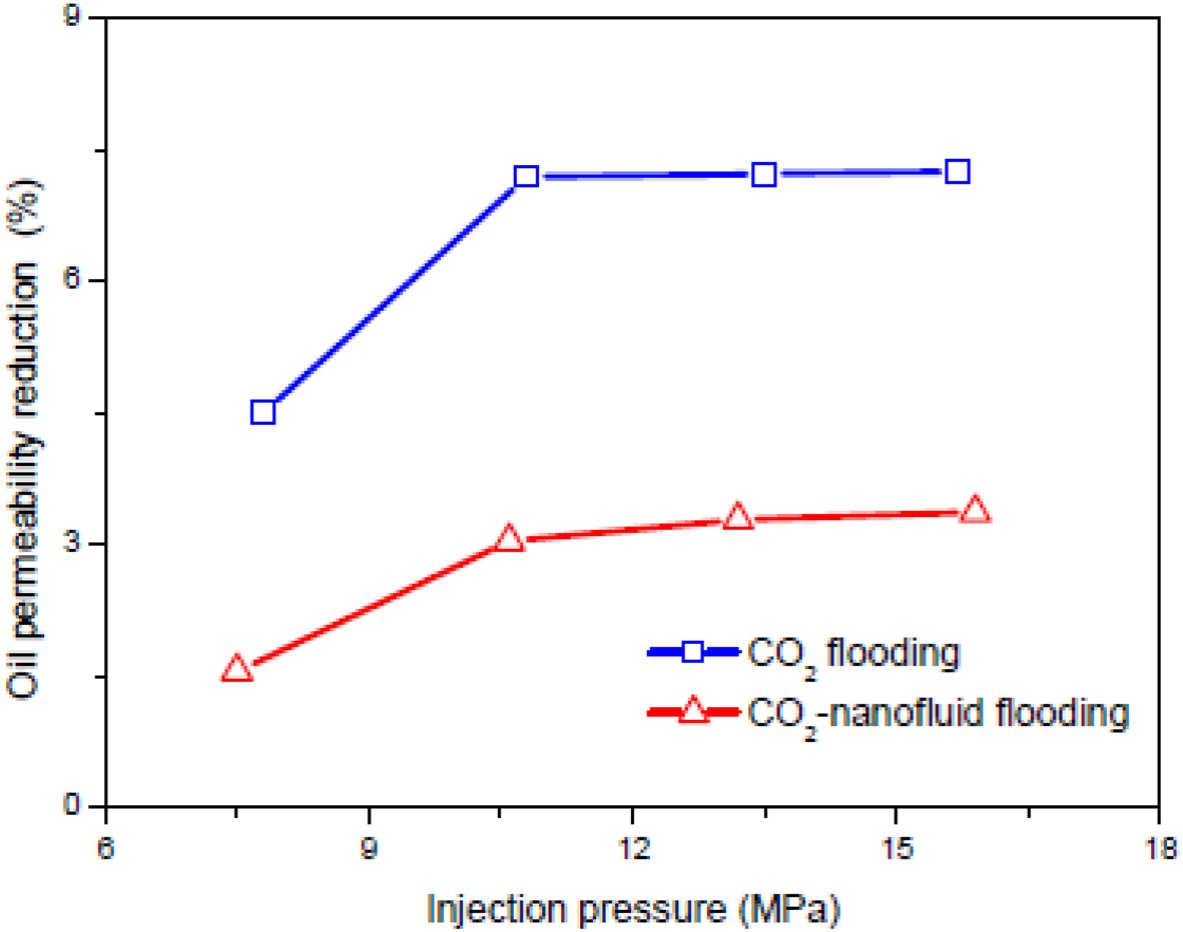
Effect of nanofluid on formation damage reduction^248^.

Hassanpour et al., investigated the effect of cobalt oxide nanoparticles on asphaltene precipitation. In this study, they used synthetic oil with a combination of asphaltene (5% (w/w)), toluene, and normal heptane and added nanoparticles with different concentrations (0.01%, 0.1% and 1% (w/w)) to the samples. They stated that with increasing pressure during the test, the IFT will decrease to a certain pressure. Then the slope of the graph changes and with a smaller slope the IFT decreases with increasing pressure. They attributed the first slope to the miscibility of carbon dioxide in crude oil and the second slope to the precipitation of asphaltene. They showed that adding nanoparticles to oil samples reduces asphaltene precipitation due to the adsorption of asphaltene by nanoparticles and prevents their aggregation. Hassanpour et al., showed that this nanoparticle increased oil production by reducing asphaltene precipitation. The results of sensitivity analysis showed that the best concentration for this nanoparticle to reduce asphaltene precipitation is 0.1% (w/w)^247^. In 2016, Lu et al., investigated the process of asphaltene adsorption by aluminum oxide nanoparticles and its effect on the amount of asphaltene deposition. In this study, they used two methods to evaluate the adsorption of asphaltene by nanoparticles. In the first part, they made synthetic oil with different concentrations of asphaltene and added nanoparticles with different concentrations. In the next part, they prepared a sample of synthetic oil with a constant concentration of asphaltene and Added different amounts of nanoparticles to each of them. In the next section, the asphaltene precipitation was examined by interfacial tension. To complete their work and study the dynamic effect of nanoparticles on reducing asphaltene deposition, the researchers alternately injected carbon dioxide and nanofluid into the cores. Given that the interfacial tension changes depend on the asphaltene arrangement at the interface of oil and carbon dioxide, after adding nanoparticles to synthetic oil, due to the adsorption of asphaltene on the interface of two phases and no aggregation in the bulk of fluid, the slope of the surface tension diagram at high pressures is reduced which indicates a decrease in asphaltene deposition in the presence of nanoparticles. They stated that solid–liquid equilibrium could be used to describe adsorption isotherms. They pointed out that the amount of nanoparticles was inversely related to the amount of asphaltene precipitation. According to the results of the dynamic test (injection of carbon dioxide and nanofluid), it can be said that the performance of nanoparticles is very promising and has been able to effectively reduce the amount of asphaltene deposition and formation damage. According to the results of sensitivity analysis on the weight percentage of nanoparticles, it was observed that 0.5% (w/w) is the best amount of nanoparticles to inhibit asphaltene precipitation and the best ratio of nanofluid to carbon dioxide was 0.1. Finally, they showed that the use of cyclic injection is less efficient than a continuous injection of nanofluids and carbon dioxide (Figs. 9 and 10)^248^.

**Figure 11:**
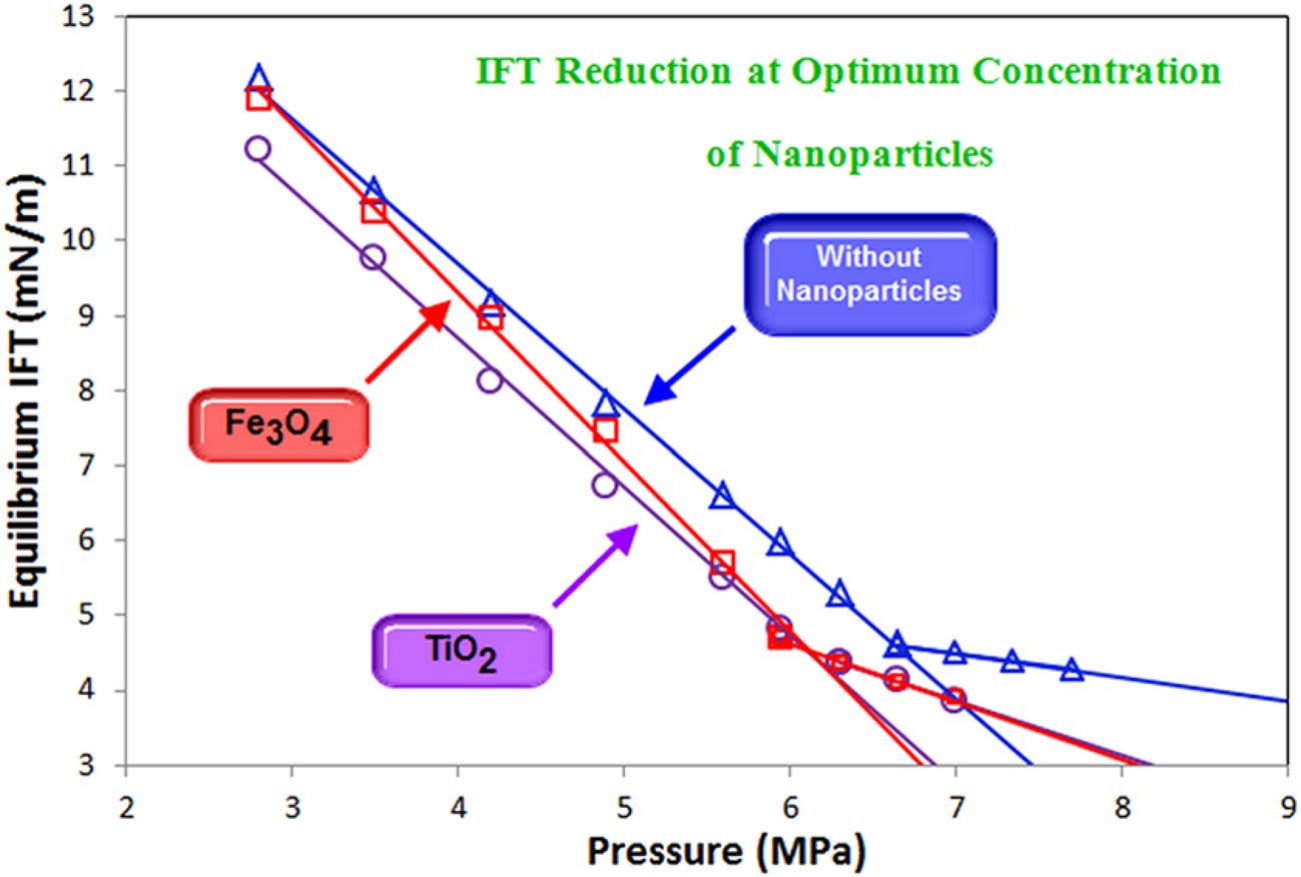
Effect of nanoparticles on asphaltene precipitation and minimum miscible pressure^249^.

Hassanpour et al., investigated the effect of two nanoparticles (iron oxide and titanium oxide) to inhibit asphaltene precipitation. They used synthetic oil in a ratio of 60–40 toluene to normal heptane. The results related to interfacial tension (change of slope of IFT vs. pressure) showed that nanoparticles were able to significantly inhibit asphaltene precipitation. Based on the results of the sensitivity analysis performed on the concentration of nanoparticles, they found that the best concentration for both nanoparticles was 1% (w/w), which caused the greatest reduction in asphaltene precipitation. The authors compared the two nanoparticles and showed that iron oxide nanoparticles performed better than titanium oxide and were able to reduce asphaltene precipitation by 18% while titanium oxide reduced precipitation by 17%. The difference between these two nanoparticles is mostly seen in the EOR-related parameters, the iron oxide nanoparticles performed better than titanium oxide and were able to improve the minimum miscible pressure more effectively (Fig. 11)^249^.

**Figure 12:**
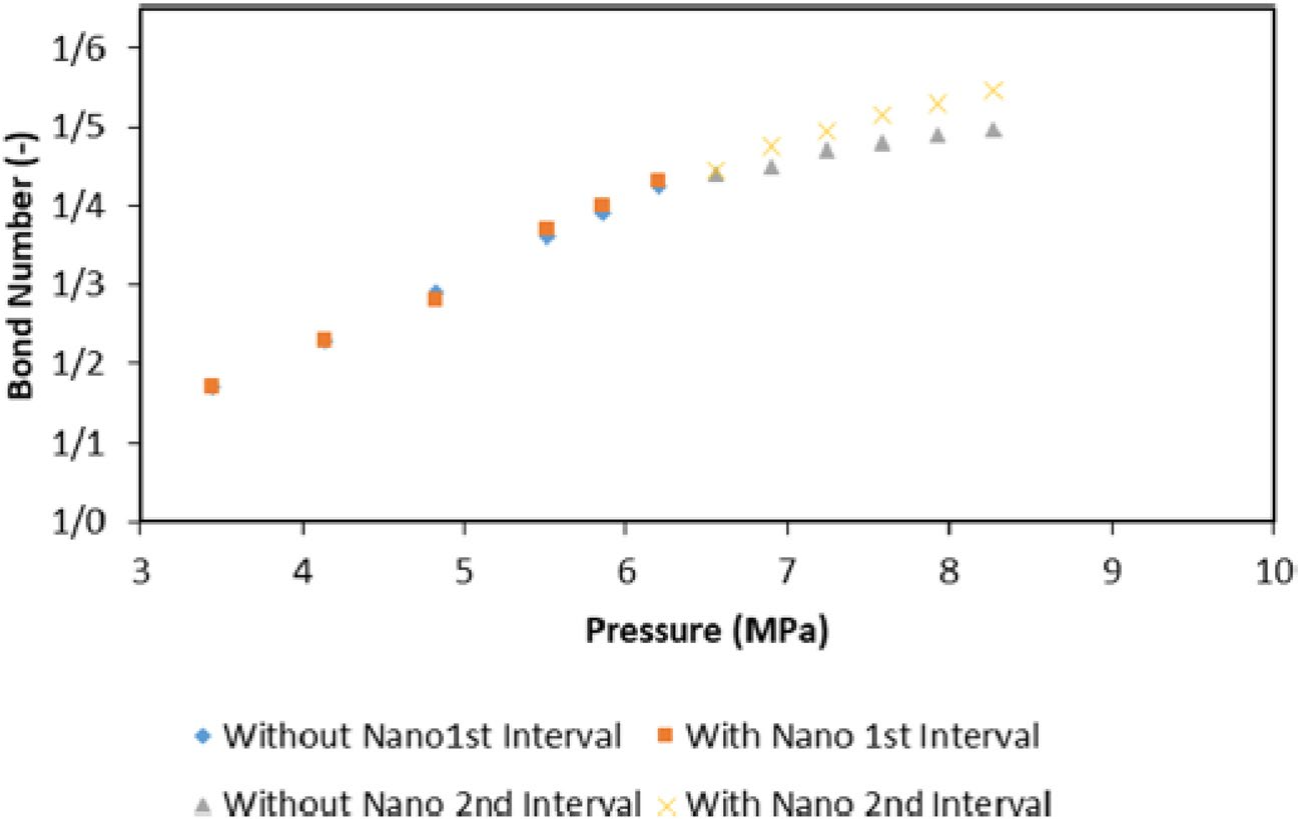
The effect of nanoparticles on bond number^250^.

In 2020, Parsai et al., studied the process of formation damage during gas injection in the presence of iron nanoparticles. In this work, they used the vanishing interfacial tension method to investigate the effect of nanoparticles on asphaltene precipitation. In this work, the authors studied the asphaltene deposition process by measuring the interfacial tension between synthetic oil and carbon dioxide by the pendant drop method. Change in the slope of the interfacial tension vs. pressure diagram in this method is the way to detect asphaltene precipitation. In this method, the diagram has two or three slopes (often two slopes), the slope of the high-pressure part is less than the slope of the low-pressure part, and this change of slope will indicate the asphaltene precipitation. The results of the Vanishing Interfacial Tension (VIT) test showed that with increasing the amount of nanoparticles in synthetic oil, the slope of the diagram increases at higher pressures and shows that the interfacial tension decreases with a higher coefficient in the presence of nanoparticles, in other words, asphaltene precipitation will decrease. In addition to the effect of nanoparticles in inhibition of asphaltene precipitation, the minimum miscible pressure was also reduced. The effect of nanoparticles on the bond number is shown in the Fig. 12^250^.

#### Dispersion of nanoparticles in the gas phase

Preventing the formation damage due to asphaltene deposition in reservoirs is critical work in the EOR processes especially the gas-based method. Hashemi et al., investigated the potential effect of nickel oxide (NiO) nanoparticles in inhibition of asphaltene deposition in a porous medium during carbon dioxide injection. Hashemi et al., used NiO nanoparticles dispersed in the gas phase by polydimethylsiloxane (PDMS) to reduce asphaltene deposition and increase the efficiency of the gas-based EOR process. Experimental results obtained from CO2 miscible injection conditions showed a significant improvement in permeability and porosity of the core, and also reduction in asphaltene aggregation in the porous medium, and an increase in the oil recovery factor after injection of gas modified with nanoparticles (Table 3)^251^.

**Table 3:** Changes in production parameters during modified gas injection^251^.

	With nanoparticles	Without nanoparticles
Change in saturation (%)	%0	0
Asphaltene deposition (g)	0.0128	0.1033
Permeability reduction (%)	2	20
porosity reduction (%)	0.24	1.2
Oil recovery (%)	78.57	71

Azizkhani and Gandmakar, because the dispersion of nanoparticles in the liquid phase has field limitations, decided on the dispersion of nanoparticles in the gas phase. The authors used carbon dioxide in the supercritical state and investigated the cloud point pressure to ensure the temperature and pressure at which the nanoparticles are dissolved in the gas phase. They also studied the effect of nanoparticles on asphaltene deposition and the minimum miscible pressure during gas injection. In the cloud point method, at first the pressure at a constant temperature sets above the critical pressure and rises to a point where the fluid inside the PVT cell becomes single-phase. Then, by reducing the pressure, we get to the point where the fluid inside the PVT cell becomes foggy. That pressure is called the minimum solubility pressure of the solid in the gas phase. In this research, they used two nanoparticles of iron oxide and aluminum oxide as direct asphaltene inhibitors. After evaluating the solubility of nanoparticles in the gas phase, they used the vanishing interfacial tension method, to investigate the effect of nanoparticles on the minimum mixing pressure. The results showed that the solubility of aluminum oxide nanoparticles is better than iron oxide and the cloud point pressure is lower for aluminum nanoparticles. However, it should be noted that solubility is one of the effective parameters, but the effect of iron oxide nanoparticles in reducing the minimum miscible pressure, and asphaltene deposition in static tests is much more effective than aluminum oxide nanoparticles. After static tests, the authors performed core flood tests to evaluate the dynamic performance of direct asphaltene inhibitors. The results showed that both nanoparticles were successful in reducing asphaltene deposition, but iron oxide nanoparticles performed better in both series of static and dynamic tests and were able to reduce asphaltene deposition more effectively^252^.

**Figure 13:**
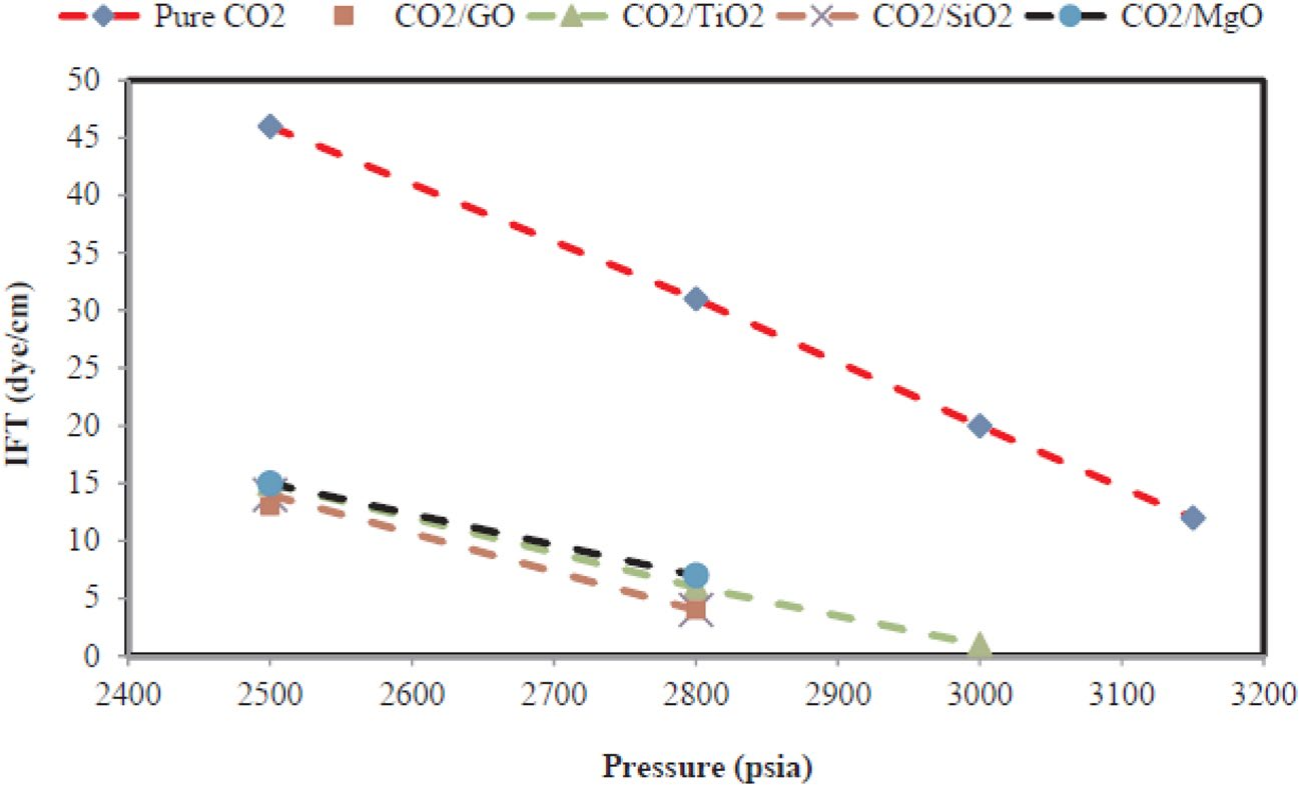
Effect of nanoparticles on IFT and MMP^253^.

**Figure 14:**
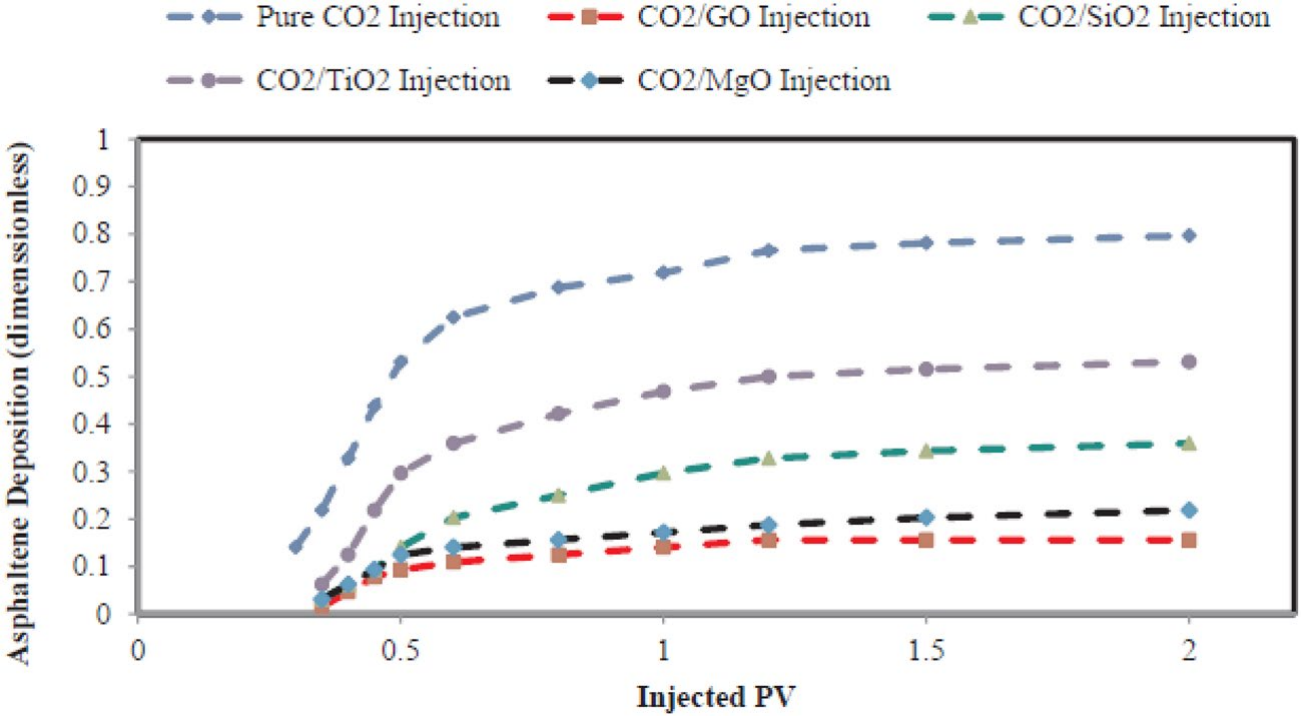
Asphaltene deposition during gas injection with/without nanoparticles^253^.

In 2020, Gandmakar and Nasriani studied direct asphaltene inhibitors during carbon dioxide injection. In this experiment, they determined the solubility and the minimum pressure required to dissolve the nanoparticles in the gas phase using the cloud point method. They studied the minimum miscible pressure by using the vanishing interfacial tension method and studied the asphaltene deposition under static conditions using a PVT cell. In this study, the authors used four nanoparticles of titanium oxide, magnesium oxide, graphene oxide, and silicon oxide. The results show that all four nanoparticles were successful in this experiment and were able to drastically reduce the amount of asphaltene deposition. Among the tested nanoparticles, graphene oxide performed better than other nanoparticles and was able to minimize asphaltene deposition. The lowest pressure for dissolution was recorded by graphene oxide and the highest pressure for titanium oxide was recorded. All nanoparticles were able to reduce MMP well, and among nanoparticles, graphene oxide showed the best performance in reducing the minimum miscible pressure. They stated that during the gas injection with nanoparticles, compared to the gas injection without nanoparticles, the deposition of asphaltene was sharply reduced, which indicates the potential of this method for use in oil fields for inhibiting asphaltene deposition. Among the nanoparticles, graphene oxide and magnesium oxide showed the best performance during gas injection. It should be noted that graphene oxide, due to its plate structure, can clog pores and cause a secondary problem after the EOR process (Fig. 13 and Fig. 14)^253^.

Given the above, the nanoparticles were able to show promising performance in asphaltene deposition inhibition and reducing MMP. However, nanoparticles dissolved in the gas phase performed better in several respects than nanoparticles dispersed in the liquid phase, including direct use as an asphaltene deposition, proper reduction of the minimum miscible pressure during gas injection, and increased oil recovery. Finally, it performs better than the cyclic injection of nanofluid and gas.

In general, gas-phase modification methods have shown promising results. By designing the appropriate material, the problems associated with the gas injection can be reduced as much as possible. This modification can increase the macroscopic efficiency and prevent asphaltene deposition, and the efficiency of the EOR process can be maximized.

## Challenges and perspectives

As stated in the previous sections, gas injection is a popular method of increasing production from oil reservoirs. Gas injection can significantly reduce the residual oil saturation by increasing the microscopic efficiency. However, the gas injection has limitations such as viscous fingering, unfavorable mobility ratio, and gravity override due to low viscosity and the difference in density compared to the reservoir fluid. In addition to these cases, gas injection can cause corrosion of pipes, problems in flow equipment, asphaltene deposition, and excessive water production. Using thickeners to increase the viscosity is one of the direct methods to overcome the low viscosity of the gas phase and control the mobility. Other mobility control methods, such as water alternating gas injection, have many problems such as: (1) reduced injectivity, (2) corrosion, (3) scale formation, (4) different temperatures of injected phases, and can affect the enhanced oil recovery process. On the other hand, foam injection to control mobility also has significant problems, such as (1) adsorption of chemicals on the reservoir rock surface, (2) foam stability, and (3) foam compatibility. These reasons increase the need to use gas thickeners. It should be noted that gas thickeners also have limitations and challenges that have overshadowed their use in the field scale. The significant drawbacks observed in studies focused on thickeners are high uncertainty in measurements, insufficient viscosity improvement, the toxicity of chemicals used, high cost, and improper solubility of materials in the gas phase. The studies conducted to reduce the mentioned problems and limitations have had promising results. It can be said that this method can be used as an alternative to indirect methods of increasing viscosity in the future.

Another problem of gas injection is asphaltene deposition in the wellbore and loss of oil production. The common methods of preventing and treating asphaltene deposition have many problems, such as (1) need for complex equipment, (2) more labor force, (3) expensive materials, (4) high operational cost, (5) non-permanence of the methods, and the possibility of re-deposition of asphaltene and low durability, and (6) indirectness of the methods and loss of part of the production. According to the reasons mentioned, using nanoparticles as a direct asphaltene inhibitor can minimize the problems caused by asphaltene deposition during gas injection. In addition, it also reduces the minimum miscible pressure and can significantly increase the efficiency of the EOR process. There have not been many studies on the effect of nanoparticles dispersed in the gas phase on asphaltene deposition, but limited studies in this field show promising results. By conducting further studies on the dispersion of nanoparticles in the gas phase, this method can minimize asphaltene deposition and increase the efficiency of the EOR process.

## Summary and conclusion

The gas injection process is one of the most popular EOR methods, but it always faces many challenges. The purpose of this study is to present the challenges of gas-based EOR processes and gas-phase modification to overcome these problems. A summary of the results obtained in laboratory and field studies is as follows:

1. Gas injection is one of the most popular methods of EOR and can increase the microscopic sweep efficiency and increase the overall production efficiency.
2. However, gas injection always has limitations that can reduce the efficiency of the EOR process and make it fail. The gas-based EOR methods have low macroscopic efficiency due to improper mobility ratio and gravity override phenomenon. This can significantly reduce the efficiency of the EOR process.
3. Another problem that gas-based EOR processes face is fluid–fluid and rock-fluid incompatibility. By injecting gas into the reservoirs, the composition of the oil changes, and this phenomenon causes the deposition of asphaltene in the porous medium.
4. To increase the efficiency of gas injection and overcome the existing problems, it is necessary to make changes to the gas phase.
5. Water alternating gas injection is one of the methods used in oil industry to overcome undesirable mobility ratio. This method has the characteristics of both water injection methods (high macroscopic efficiency) and gas injection (high microscopic efficiency). Another method used in industrial studies is foam injection instead of gas injection into oil reservoirs, which has yielded good results.
6. Another way to increase the gas phase viscosity is to use thickeners. These materials by increasing the viscosity of the gas phase, prevent the viscous fingering phenomenon and early gas breakthrough, and increase the sweep efficiency. The studies that have been done so far include problems such as the toxicity of the materials, the uncertainty in the use of this technology, the high cost, and insufficient increase in viscosity. For this reason, no field application has been reported for this method, but the proper design of thickeners can be overcome this problem.
7. To overcome asphaltene deposition during gas injection and reduce the minimum miscible pressure, the researchers suggested the use of nanoparticles. Studies on the use of nanoparticles are divided into two categories: nanoparticles soluble in the liquid phase and nanoparticles soluble in the gas phase. The results showed that the nanoparticles could control asphaltene deposition and reduce the minimum miscible pressure.
8. Regarding the use of nanoparticles, it should be noted that the solubility of nanoparticles in the liquid phase (oil) is not applicable in oil fields. However, the dispersion of nanoparticles in the gas phase as a direct inhibitor of asphaltene has the potential to be used in the oil field and, in addition to reducing asphaltene deposition, reduces the minimum miscible pressure.

## Data Availability

All data generated or analyzed during this study are included in this published article.
